# Medication use during pregnancy and the risk of gastroschisis: a systematic review and meta-analysis of observational studies

**DOI:** 10.1186/s13023-023-02992-z

**Published:** 2024-01-30

**Authors:** Silvia Baldacci, Michele Santoro, Lorena Mezzasalma, Anna Pierini, Alessio Coi

**Affiliations:** 1 Unit of Epidemiology of Rare Diseases and Congenital Anomalies, Institute of Clinical Physiology, National Research Council, Via G. Moruzzi 1, 56124 Pisa, Italy; 2 Fondazione Toscana Gabriele Monasterio, Pisa, Italy

**Keywords:** Gastroschisis, Medication, Systematic review, Meta-analysis, Risk factors, Observational studies

## Abstract

**Objectives:**

The aetiology of gastroschisis is considered multifactorial. We conducted a systematic review and meta-analysis to assess whether the use of medications during pregnancy, is associated with the risk of gastroschisis in offspring.

**Methods:**

PubMed, EMBASE, and Scopus were searched from 1st January 1990 to 31st December 2020 to identify observational studies examining the association between medication use during pregnancy and the risk of gastroschisis. The Newcastle–Ottawa Scale was used for the quality assessment of the individual studies. We pooled adjusted measures using a random-effect model to estimate relative risk [RR] and the 95% confidence interval [CI]. I^2^ statistic for heterogeneity and publication bias was calculated.

**Results:**

Eighteen studies providing data on 751,954 pregnancies were included in the meta-analysis. Pooled RRs showed significant associations between aspirin (RR 1.66, 95% CI 1.16–2.38; I^2^ = 58.3%), oral contraceptives (RR 1.52, 95% CI 1.21–1.92; I^2^ = 22.0%), pseudoephedrine and phenylpropanolamine (RR 1.51, 95% CI 1.16–1.97; I^2^ = 33.2%), ibuprofen (RR 1.42, 95% CI 1.26–1.60; I^2^ = 0.0%), and gastroschisis. No association was observed between paracetamol and gastroschisis (RR 1.16, 95% CI 0.96–1.41; I^2^ = 39.4%).

**Conclusions:**

These results suggest that the exposure in the first trimester of pregnancy to over the counter medications (OTC) such as aspirin, ibuprofen, pseudoephedrine and phenylpropanolamine as well as to oral contraceptives, was associated with an increased risk of gastroschisis. However, these associations are significant only in particular subgroups defined by geographic location, adjustment variables and type of control. Therefore, further research is needed to investigate them as potential risk factors for gastroschisis, to assess their safety in pregnancy and to develop treatment strategies to reduce the risk of gastroschisis in offspring.

PROSPERO registration number: CRD42021287529.

**Supplementary Information:**

The online version contains supplementary material available at 10.1186/s13023-023-02992-z.

## Introduction

Gastroschisis is a rare congenital anomaly of the abdominal wall where part of the large intestine, small intestine and rarely other abdominal organs protrude through the right side in the ventral abdomen. This anomaly does not involve the umbilical cord, and the bowel herniation is not covered by a membrane [1, 2]. Gastroschisis is mainly an isolated congenital anomaly [3].

Gastroschisis is a severe congenital anomaly with a high impact on affected individuals and their families regarding the quality of life and healthcare service needs, representing a public health issue [4–6]. Identifying potential risk factors for gastroschisis is a public health priority aimed at developing preventive actions to reduce this congenital anomaly's prevalence and health burden.

Clinical and embryological studies demonstrated that the wall defect, results from either an amniotic rupture or a separation of the amnio-ectodermal junction at the pars flaccida, with the midgut prolapse into the amniotic cavity. The rupture occurs at the right side of the umbilical cord, during the normal physiologic herniation. Moreover, through the observation of embryonic development events has been estimated that gastroschisis occurs between 56 and 77 days post conception. [1, 2, 7].

The aetiology of gastroschisis is still unclear, most likely multifactorial, caused by the interaction of genes and environmental risk factors.

Several studies reported an increasing prevalence rate worldwide over the past decades, most of them with a higher prevalence among young women aged less than 20 years [8–12]. While epidemiological studies have consistently evidenced the strong association between gastroschisis and young maternal age, the aetiologic role of environmental factors is still under investigation [13–15].

Three previous literature reviews collected observational studies assessing the possible associations between non-genetic risk factors (e.g., lifestyle, socio-demographic, maternal illness, medication use) and gastroschisis with widely divergent results [16–18].

For medication exposure during pregnancy, the observational studies suggested an increased risk of gastroschisis among pregnant women who have used aspirin, ibuprofen, and decongestants. At the same time, inconsistent results were found for anti-histamines, antibiotics and oral contraceptives [16–18].

A systematic review with meta-analysis by Kozer et al. (2002) on maternal aspirin use during pregnancy and congenital anomalies showed that the exposure to aspirin during the first trimester was associated with a significant increased risk of gastroschisis [19]. A recent meta-analysis showed that maternal smoking, illicit drug use, and alcohol consumption during early pregnancy are associated with an increased risk of gastroschisis in offspring [20].

The present study aimed to qualitatively and quantitatively synthesize the available epidemiological evidence to investigate the association between medication use during pregnancy and gastroschisis.

## Methods

### Registration of the review protocol

The protocol of this study was registered in PROSPERO (International Prospective Register of Systematic Reviews, no. CRD42021287529), available at the website: https://www.crd.york.ac.uk/prospero/.

Due to the nature of the study, neither ethics approval nor informed consent was required.

### Literature search strategy

PubMed, EMBASE, and Scopus databases were searched electronically, from January 1st 1990 to December 31st 2020, for all observational studies examining the association between medication exposure in pregnancy and the risk of gastroschisis. For the search strategy, we used the following combinations of the relevant Medical Subject Headings (MeSH) and keywords related to the exposure and the outcome of interest: [maternal AND “medication” OR "medical drug” OR “drug therapy”] AND gastroschisis. Additional studies were manually searched by reference lists of the relevant papers. We searched English language and human studies only.

Details of the search strategy are presented (see Additional file 1: Table S1).

The systematic review and meta-analysis were conducted following the Preferred Reporting Items for Systematic reviews and Meta-Analyses (PRISMA) guidelines [21].

### Inclusion and exclusion criteria

Studies were included if they were observational studies with cohort, case–control or nested case–control design, reporting a comparison between pregnant women who had been exposed to one or more medications and women who had not been exposed to any medication during pregnancy and outcomes that included gastroschisis. The studies that provided estimates of the association and their corresponding 95% confidence intervals [CIs] or presenting sufficient data to estimate them were included. Live births, stillbirths, and terminations of pregnancy could all be considered suitable endpoints for pregnancies.

Animal studies, cross-sectional studies, systematic reviews, meta-analyses, reviews, letters, editorials, reports, comments, documents issued by regulatory bodies, and book chapters were excluded. Those studies that investigated postnatal maternal and/or infant exposure to medicines were also excluded.

No inclusion or exclusion criteria concerned the timeframe of pregnancy exposure to the medicines.

### Data extraction and quality assessment

**Fig. 1:**
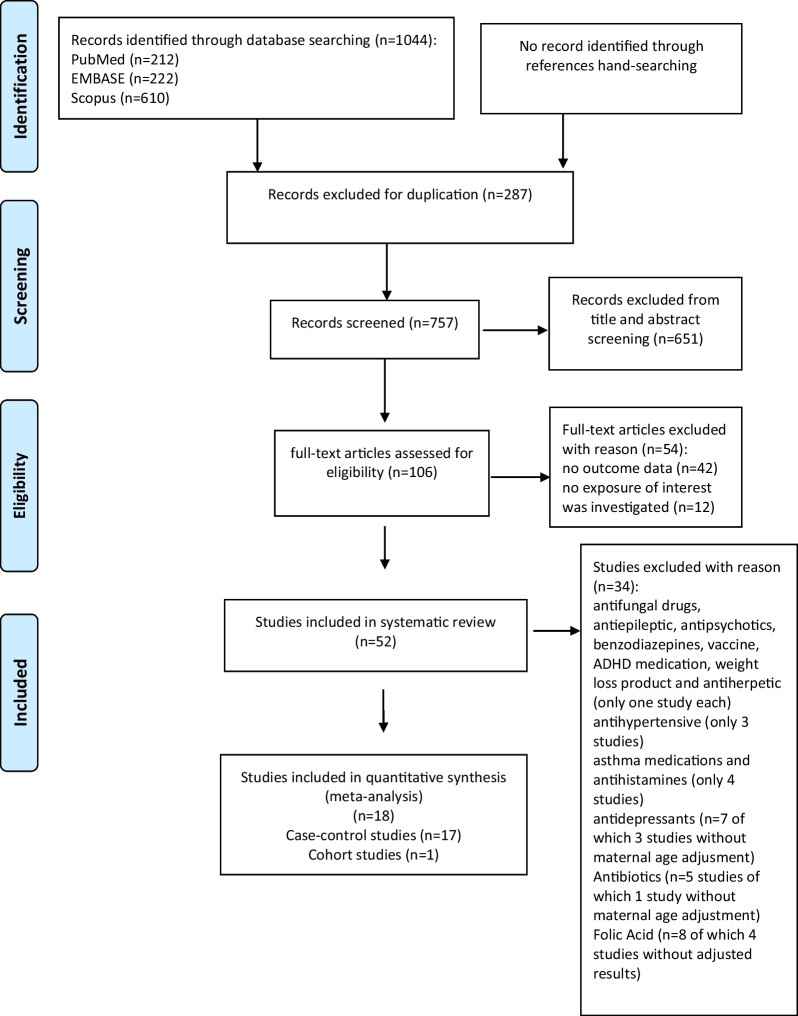
Literature search PRISMA flow diagram

Figure 1 shows the process of the articles identification and inclusion. Among 1044 papers identified from the literature, 287 duplicative papers were removed. Two couple of authors (SB and AC; MS and LM) reviewed the remaining 757 articles. Each couple screened titles and abstracts of the half of the 757 articles, independently to assess conformity with inclusion criteria; 651 articles were excluded because were irrelevant to the current systematic review. Disagreement regarding potential relevance was resolved by discussion between the reviewers within the same couple. Next, each reviewer independently examined the full-text of the remaining 106 articles to assess eligibility, according to the inclusion criteria; 52 articles were considered eligible and were included in the qualitative synthesis. Among them, only 18 articles fitted with our meta-analysis criteria. Disagreements on the inclusion eligibility were resolved by discussion between the reviewers within the same couple.

Each reviewer independently extracted data from included studies, using a standardised form reporting: first author, year of publication, study site, study design, study period, data source, sample size, type of exposure, exposure definition, exposure assessment, window of exposure, adjusted or unadjusted measures of association (odds ratio [OR], risk ratio [RR], hazard ratio [HR] according to the study design) and associated 95% confidence intervals [CIs] and details of the confounders that were adjusted for.

Meta-analysis was performed only if more than five studies were available for a class of medications and for a specific agent, adjusted at least for maternal age.

Quality assessment of the studies was performed independently by each reviewer using the Newcastle–Ottawa Scale (NOS) [22] (available at https://www.ohri.ca/programs/clinical_epidemiology/oxford.asp), which is recommended by the Agency for Healthcare Research and Quality (AHRQ), and the checklists are provided (see Additional file 1: Tables S2 a, b).

The quality assessment scale was based on the following three categories: the selection category ranged from 0 to 4 stars, the comparability category ranged from 0 to 2 stars, and the exposure category ranged from 0 to 3 stars. Therefore, the overall score range was from 0 to 9 stars. For the comparability category, controlling for maternal age was considered the most important factor and was given 1 star to the study controlling for this factor. If any other factors (e.g., lifestyle habits, socioeconomic status (SES), demographic factors) were controlled for, they received 2 stars.

To assess methodological issues that were not common to case–control studies and cohort studies, we used the following criteria: case–control studies reported participation rates with a different of < 5% (1 star); cohort studies with subjects lost to follow up < 95% (1 star), and > 95% or not statement (0 star).

As several literature showed [23–28], we considered the studies that scored from seven to nine stars as good quality, those that scored six or five stars as medium quality, and those that scored less than five as poor quality (see Additional file 1: Table S4).

### Statistical analyses

The pooled RR and the 95% CI were calculated using a random-effect model. Individual study estimates were log-transformed before the generation of the pooled estimate. We investigated the pooled RR for gastroschisis with users of medication in pregnancy compared with non-users. The presence of heterogeneity was examined by the Higgins I^2^ test, and the p-value less than 0.05 was considered statistically significant for heterogeneity [29].

Furthermore, we performed subgroup analyses defined by geographic location, adjustment variables, exposure period and type of controls. Additionally, to assess the robustness of the results, we conducted a sensitivity analysis excluding the study with the highest weight, with a NOS < 7 and those studies published before 1999.

Potential publication bias was evaluated visually by Funnel Plot and, more formally, by Egger’s test (significance level was set at p < 0.1) [30, 31]. We corrected potential publication bias using the trim-and-fill method to provide bias-adjusted results [32, 33].

Statistical analyses were performed using Stata SE version 16.0 (StataCorp LP, College Station, Texas).

## Results

### Literature search results

Fifty-two studies fitted against the inclusion criteria and were eligible for the qualitative synthesis as specified in the PRISMA flow diagram (Fig. 1). Among these, thirty-four studies [11, 12, 34–45, 47–66] failed meta-analysis inclusion criteria (Fig. 1). Detailed characteristics of these studies are described (see Additional file 1: Table S3). Eighteen studies [14, 15, 46, 67–81], fitting meta-analysis requisites, were included in the meta-analysis providing data on 751,954 pregnancies.

### Description of the included studies

Detailed characteristics of the 18 individual studies included in the meta-analysis are provided (Table 1).

**Table 1:** Overview of studies included in the systematic review and meta-analysis (listed alphabetically by the first author)

Study, year, country	Study design/time/data source/case ascertainment	Sample^a^ size	Exposure	Exposure definition	Exposure assessment	Window of exposure	Measures of effect (95% CI)	Adjusted variables	NOS score^b^
Draper et al. [46] United Kingdom	Multicentre Case–Control (matched by maternal age, place of delivery, residence)/2001–2003/regional congenital anomalies registries/LB	144 Gastroschisis cases; 432 controls	Aspirin	Use, non-use	Maternal interview	First trimester	Aspirin use aOR 20.4 (2.2–191.5)	Maternal age, BMI, marital status, aspirin use, smoking, recreational drug use, vasoconstrictive recreational drug use, gynecologic infection/disease, homeowner	9
Feldkamp et al. [67] United States	Multicentre Case–control/1997–2004/birth defects surveillance systems, birth certificates or hospitals birth logs (NBDPS)/LB, SB, ET	11,610 cases; 4500 controls Gastroschisis cases:531	Acetaminophen	Use, non-use	Computer assisted telephone interview	First trimester	Acetaminophen use aOR 1.03 (0.83–1.28)	Maternal age, BMI education, gestational diabetes, fever, smoking, folic acid use, race/ethnicity, parity	8
Freitas et al. [68] Brasil	Case–control (matched by maternal age, preconception BMI and gestational age)/2013–2015/ ultrasound scan/LB	57 Gastroschisis cases; 114 controls	Any medication Oral contraceptives	Use, non-use	Questionnaire	One month before to third months after conception	Any Medications aOR 1.47 (0.77–2.78) Oral contraceptives aOR 1.47 (0.67–3.25)	Maternal age, preconception BMI, gestational age	6
Given et al. [69] Europe	Multicentre Case–control/1995–2012/EUROmediCAT registries/LB, SB, ET	1587 Gastroschisis cases; 153,357 controls	See the original article	Use, non-use	Registries, maternity records, medical prescriptions, maternal interviews	First trimester of pregnancy	See the original article for a reproduction of the original results table	Maternal age, registry, time period	6
Goodman et al. [70] United States	Case–control (matched for maternal age and race/ethnicity)/2010–2012/ultrasound scan/LB	31Gastroschisis cases; 76 controls	Oral contraceptive Over the counter (OTC) Aspirin Ibuprofen	Use, non-use	Maternal interview	One month before to or during pregnancy	Oral contraceptive aOR 0.83 (0.29–2.85) Any OTC aOR 1.02 (0.34–3.39) Aspirin aOR 0.63 (0.01–6.84) Ibuprofen aOR 1.25 (0.36–3.91)	Maternal age, race/ethnicity, registry, time period, insurance, education, low BMI, nulliparity	6
Mac Bird et al. [15] United States	Multicentre Case–control/ 1997–2003/birth defects surveillance system, birth certificates or hospital discharge records/(NBDPS)/LB, SB, ET	653 cases; 4967 controls Gastroschisis cases:485	Aspirin Ibuprofen Acetaminophen Pseudoephedrine	Use, non-use	Computer-assisted telephone interviews	One month before conception through 3 months postconception	Aspirin aOR 1.25 (0.77–2.05) Ibuprofen aOR 1.61 (1.23–2.10) Acetaminophen aOR 0.93 (0.72–1.19) Pseudoephedrine aOR 1.00 (0.66–1.51)	Maternal age, race/ethnicity, BMI, sex alcohol, smoking, drug, parity, family income, aspirin,, pre-existing gestational diabetes pseudoephedrine, ibuprofen, naproxen acetaminophen, study center, folic acid use,	8
Martinez-Frias et al. [71] Spain	Case–control (matched by sex and birth hospital)/1976–1996/Spanish Collaborative Study of Congenital Malformations (ECEMC) hospital-based and surveillance system/LB	45 Gastroschisis cases; 690 controls	Salicylates	Use, non-use	Questionnaire	First trimester	45 GS cases and 44 paired controls aOR 2.63 (0.41–20.87) 45 GS cases and 690 controls aOR 3.47 (1.27–9.49)	Maternal age, smoking	6
Raitio et al. [72] Finland	Case–control (matched by maternal age, residence and time of conception)/2004–2014/Finnish Register of congenital Malformations, Medical birth registry, Register of Induced abortions and the Care Register of Health Care/LB	188 Gastroschisis cases; 919 controls	Pseudoephedrine Non-steroidal anti-inflammatory drugs (NSAIDs)	Use, non-use	Register of Reimbursed Drug Purchases	First trimester	Pseudoephedrine aOR 10.0 (0.91–110) NSAIDs aOR 0.72 (0.38–1.39)	Maternal age, residence, time of conception	6
Rebordosa et al. [73] Denmark	Cohort study/1996–2003/Danish National Birth Cohort (DNBC)	88,142 liveborn singletons Gastroschisis cases:12	Acetaminophen use	Exposed: use of drugs containing acetaminophen at least once No exposed: no exposure to acetaminophen	Computer-assisted follow-up telephone interviews self-administered questionnaire at enrollment	First trimester	Acetaminophen aHR 0.91 (0.55–4.09)	Mother’s age, birth year, birth order, child’s gender, history of chronic diseases	7
Robledo-Aceves et al. [74] Mexico	Case–control (matched for gender)/2009–2013/Centro de Registro y Investigación sobre Anomalías Congénitas (CRIAC), hospital-based active birth defect monitoring program/LB	90 Gastroschisis cases; 180 controls	Paracetamol Aspirin Ibuprofen Hormonal contraceptives	Use, non-use	Mother Interview	First trimester	Paracetamol aOR 0.7 (0.3–1.4) Aspirin aOR 8.6 (0.8–89.1) Ibuprofen aOR 0.2 (0.0–2.1) Hormonal contraceptives aOR 3.7 (1.0–13.0)	Maternal age, alcohol consumption, anemia during pregnancy, pre-pregnancy BMI < 18.5 kg/m^2^, first-trimester tobacco smoking, and passive tobacco smoking	6
Torfs et al. [75] United States	Multicentre Case–control (matched by maternal age)/1988–1990/ California Birth Defects Monitoring Program registry (CBDMP), birth records of the California Department of Vital Statistics/LB	110 Gastroschisis cases; 220 controls	Vasoconstrictors Aspirin Ibuprofen Decongestants Acetaminophen Pseudoephedrine Phenylpropanolamine Aspirin or Ibuprofen	Use, non-use	Maternal interview	First trimester	Aspirin or Ibuprofen aOR 4.55 (1.40–14.73) Decongestants aOR 2.37 (0.76–7.38) Aspirin aOR 4.67 (1.21–18.05) Ibuprofen aOR 4.0 (1.00–15.99) Acetaminophen aOR 1.0 (0.59–1.69) Pseudoephedrine aOR 2.10 (0.80–-5.49) Phenylpropanolamine aOR 10.0 (1.17–85.59)	Maternal age	7
Waller et al. [76] United States	Multicentre Case–control/ 1997–2003/birth defects surveillance systems, birth certificates or hospitals birth logs (NBDPS)/LB, SB, ET	9986 cases; 4000 controls Gastroschisis cases:447	Oral contraceptives	Use, non-use	Computer assisted telephone interview	Three months before conception and during the first trimester	Last Used 2–3 Months Before Conception aOR 1.08 (0.71–1.63) Last Used 1 Month Before Conception aOR 1.19 (0.77–1.84) Used in First 3 Months aOR 1.82 (1.25–2.67)	Maternal age	7
Werler et al. [77] United States	Multicentre Case–control/ 1997–2011/birth defects surveillance systems, birth certificates or hospitals birth logs (NBDPS)/LB, SB, ET	1261 Gastroschisis cases; 10,682 controls	Aspirin Ibuprofen Oral contraceptives	Use, non-use	Computer assisted telephone interview	One month before conception through the third month of pregnancy	Aspirin aOR 1.1 (0.9–1.5) Ibuprofen aOR 1.4 (1.2–1.6) Oral contraceptives aOR 1.3 (1.1–1.6)	Maternal age, fever, injury, genitourinary infection, anti-herpetic use, alcohol, smoking, illicit drug use, bronchodilator use, contraceptive use, opioid use, inter-pregnancy interval of less than 12 months, residential move, venlafaxine, aspirin, paroxetine, ibuprofen use	7
Werler et al. [14] United States	Multicentre Case–control (matched by age and state of residence)/1997–2003/birth defects surveillance system, birth certificates or birth hospitals	514 Gastroschisis cases; 3277 controls	NSAIDs Decongestants Aspirin	Use, non-use	Computer assisted telephone interview	Two weeks before through 14 weeks after the last menstrual period	NSAIDs aOR 1.4 (1.1–1.7) Decongestants aOR 1.0 (0.7–1.4) Aspirin aOR 1.1 (0.7–1.7)	Maternal age, state of residence by stratification and for race/ethnicity, BMI, education, alcohol use, oral contraceptive use, folic acid use	7
Werler et al. [78] United States, Canada	Multicentre Case–control (matched by age)/1995–1999/medical records of 29 tertiary centre hospitals/LB	332 cases; 797 controls Gastroschisis cases:20	Vasoconstrictive Drugs	Use, non-use	Maternal interview	First 10 weeks of gestation	Vasoconstrictive Drugs aOR 1.7 (1.0–2.7)	Maternal age, drug use, alcohol use, education, smoking, income, use of acetaminophen, aspirin, antihistamines, marijuana nonsteroidal anti-inflammatory, guaifenesin	5
Werler et al. [79] United States, Canada	Multicentre Case–control (matched by age)/1995–1999/medical records of 29 tertiary centre hospitals/LB	332 cases; 798 controls Gastroschisis cases: 206	Pseudoephedrine, Phenylpropanolamine Aspirin, Ibuprofen, Acetaminophen,	Use, non-use	Maternal interview	First trimester	Pseudoephedrine aOR 1.8 (1.0–3.2) Phenylpropanolamine aOR 1.2 (0.5–3.1) Aspirin aOR 2.7 (1.2–5.9) Ibuprofen aOR 1.1 (0.7–1.8)	Maternal age, education, income, medication use, illness, illicit drug use, and cigarette smoking	6
Werler et al. [80] United States, Canada	Multicentre Case–control/1976–1990/Slone Epidemiology Unit Birth Defect Study (BDS), hospital records/LB	76 Gastroschisis cases; 2142 controls	Salicylates Ibuprofen, Acetaminophen Pseudoephedrine, Phenylpropanolamine Contraceptives	Use, non-use	Maternal interview	First trimester	Salicylates aRR 1.6 (0.9–2.7) Ibuprofen aRR 1.3 (0.4–3.7) Acetaminophen aRR 1.7 (1.0–2.9) Pseudoephedrine aRR 3.2 (1.3–7.7) Phenylpropanolamine aRR 1.5 (0.4–5.4) Contraceptives oral aOR 1.3 (0.5–3.5) Spermicides aOR 1.2 (0.5–2.9)	Maternal age, years of education, alcohol, each of the study medications, influenza, interview year, study center	6
Yau et al. [81] United States, Canada	Multicentre Case–control/1993–2010/Slone Epidemiology Center Birth Defects Study (BDS)/LB	12,734 cases; 7606 controls Gastroschisis cases:258	Intranasal decongestant only Imidazoline derivates only Pseudoephedrine, phenylephrine, phenylpropanolamine oxymetazoline, xylometazoline	Unexposed; Likely exposed; Possibly exposed in a given trimester; Exposed only outside a given trimester	Maternal interview	First trimester	Oral Decongestants aOR 1.7 (1.0–2.9) Pseudoephedrine aOR 1.5 (0.8–2.8)	Maternal age, smoking prepregnancy weight, educational level	7

*CI* confidence interval *aOR* adjusted odds ratio *aHR* adjusted hazard ratio *aRR* adjusted rate ratio *BMI* body mass index *NOS* Newcastle–Ottawa Scale *NBDPS* National Birth Defects Prevention Study *LB* live births* SB* still births* ET* elective termination of pregnancy

^a^Sample size represents the number of pregnancy episodes

^b^The quality assessment of observational studies was based on the NOS score (range 0–9 stars) obtained from three criteria: selection (range 0–4 points); comparability (range 0–2 stars) and exposure (range 0–3 stars)

Seventeen were case–control studies [14, 15, 46, 67–81] and 1 cohort study [73], comparing 26,436 gestational users to 725,518 non-users.

Overall, the studies included 28,817 cases and 723,717 controls, and they were carried out between 1992 and 2020. Eleven studies were conducted in North America (United states of America (USA) and Canada [14, 15, 67, 70, 75–81], 5 in Europe [46, 69, 71–73], 1 in South America [68] and 1 in Mexico [74].

Outcome data of 5 case–control studies originated from USA population-based surveillance registries (i.e. National Birth Defects Prevention Study) [14, 15, 67, 76, 77]; one from the birth defects registry (i.e. California Birth Defects Monitoring Program Registry) [75] and one from medical records [70]. For 4 case–control studies [78–81], outcome data were ascertained from North American hospital registries (i.e. Slone Birth Defect Study), while the outcome data of 2 studies were from the hospitals of Central America (Mexico) and South America (Brazil), respectively [68, 74]. Three case–control studies [46, 69, 72] were conducted in Europe using population-based registries; in one study, gastroschisis was ascertained using hospital records and a surveillance registry [71]. Data of the cohort study originated from the Denmark birth registry [73].

In fourteen studies, control groups were healthy newborns (i.e., without birth defects) [14, 15, 46, 67, 68, 70–77, 81]; in 2 studies the control groups included malformed and no malformed infants [78, 79] while, for 2 studies, only malformed controls were used [69, 80].

For all case–control studies, the exposure ascertainment was collected retrospectively; for the cohort study, the exposure assessment was collected prospectively. Only 1 study assessed the dose and the exact time/frequency and/or duration of medication use [14].

In 10 studies [14, 15, 67, 77–81] medications were coded using the Slone Drug Dictionary [82], in 3studies [69, 71, 72] according to the Anatomical Therapeutic Chemical (ATC) classification [83]. Other studies used self-report questionnaires and/or medical records or filled prescriptions [46, 68, 70, 73, 74].

In 13 studies [46, 47, 69–75, 77–81], the exposure window was the first trimester of pregnancy; for 5 studies [14, 15, 68, 76, 77], the exposure period was one month before conception through the third month after conception (i.e. periconceptional period).

Three studies presented results adjusted for maternal age only [69, 75, 76]; 2 studies also adjusted for lifestyle habits [56, 71]; 3 studies for maternal age and other additional factors (i.e. lifestyle habits or socioeconomic status (SES) factors) [68, 72, 73] and 10 studies adjusted for maternal age, lifestyle habits, SES factors and others additional factors (Table 1) [14, 15, 46, 67, 70, 77–81].

According to the NOS quality assessment, 9 studies were classified as good quality (NOS score ≥ 7) and 9 as medium quality studies (4 < NOS score < 7).

## Results of meta-analysis

### Aspirin

**Fig. 2:**
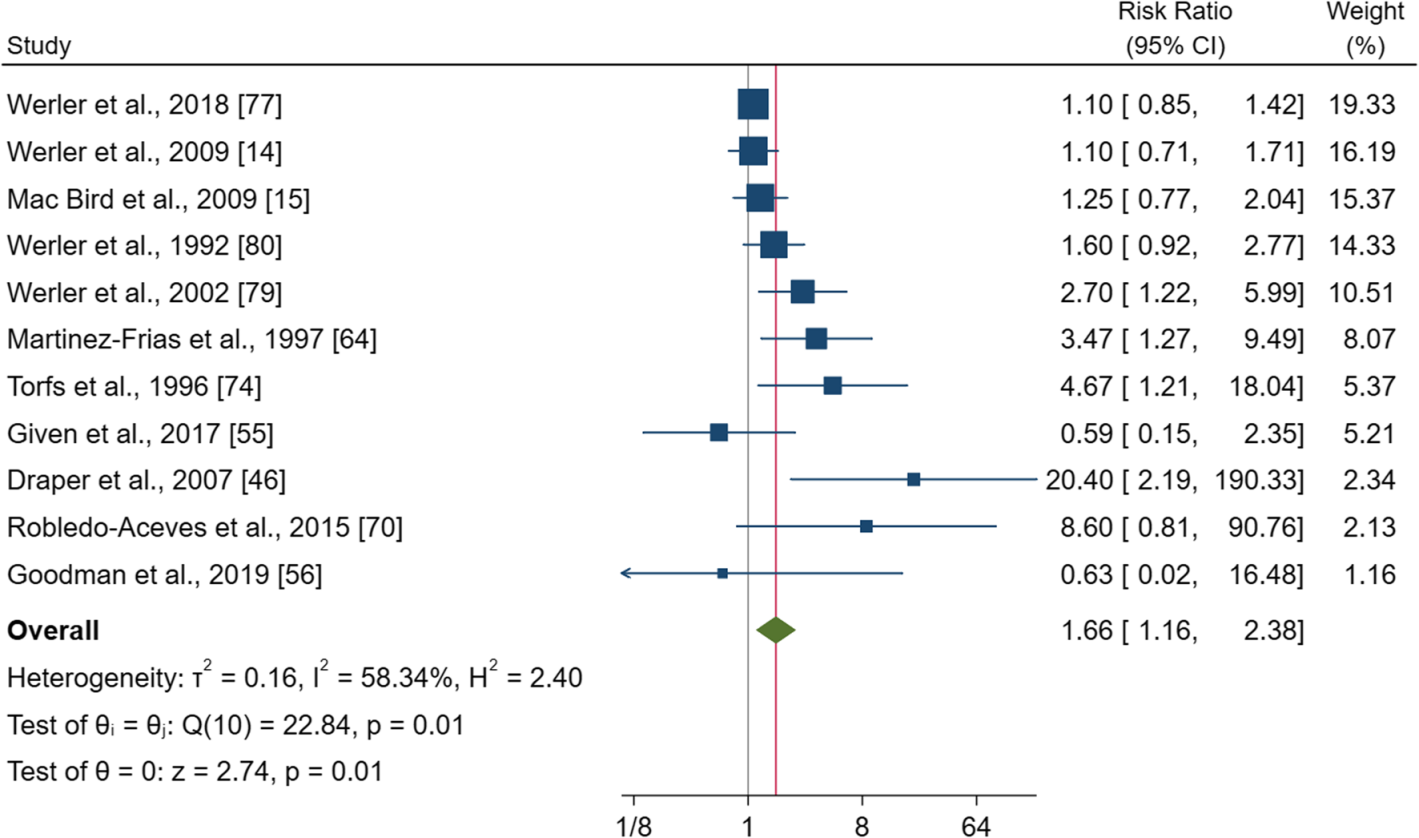
Forest plot (random-effect analysis) of the association between aspirin use during pregnancy and the risk of gastroschisis

Eleven studies comprising 181,357 pregnancies were included in the meta-analysis for aspirin use. The pooled effect estimate showed a significantly increased risk of gastroschisis with a RR of 1.66 (95% CI 1.16–2.38, p = 0.01) (Fig. 2). There was evidence of heterogeneity between study (I^2^ = 58.3%; p = 0.01). Subgroup analysis showed significant increases for women living in North America and taking aspirin during the first trimester [RR = 1.33 (95% CI 1.04–1.70), p = 0.021; RR = 2.48 (95% CI 1.43–4.33), p = 0.001]. Moreover, the subgroups of fully adjusted studies, and those where the control group included newborns with no birth defects, had statistically significant RRs (Additional file 1: Fig. S7). The sensitivity analysis confirmed an increased risk of gastroschisis, even if the CI was significance when only the studies with a high-quality score or those more recently published were included. Visually, the funnel plot showed some degree of asymmetry with a larger number of studies favouring the effect (see Additional file 1: Fig. S7a), and the Egger’s test confirmed this asymmetry (p = 0.00). However, the trim-and-fill procedure imputed 2 studies and suggested a correct RR of 1.51 (95% CI 1.04–2.20).

### Ibuprofen

**Fig. 3:**
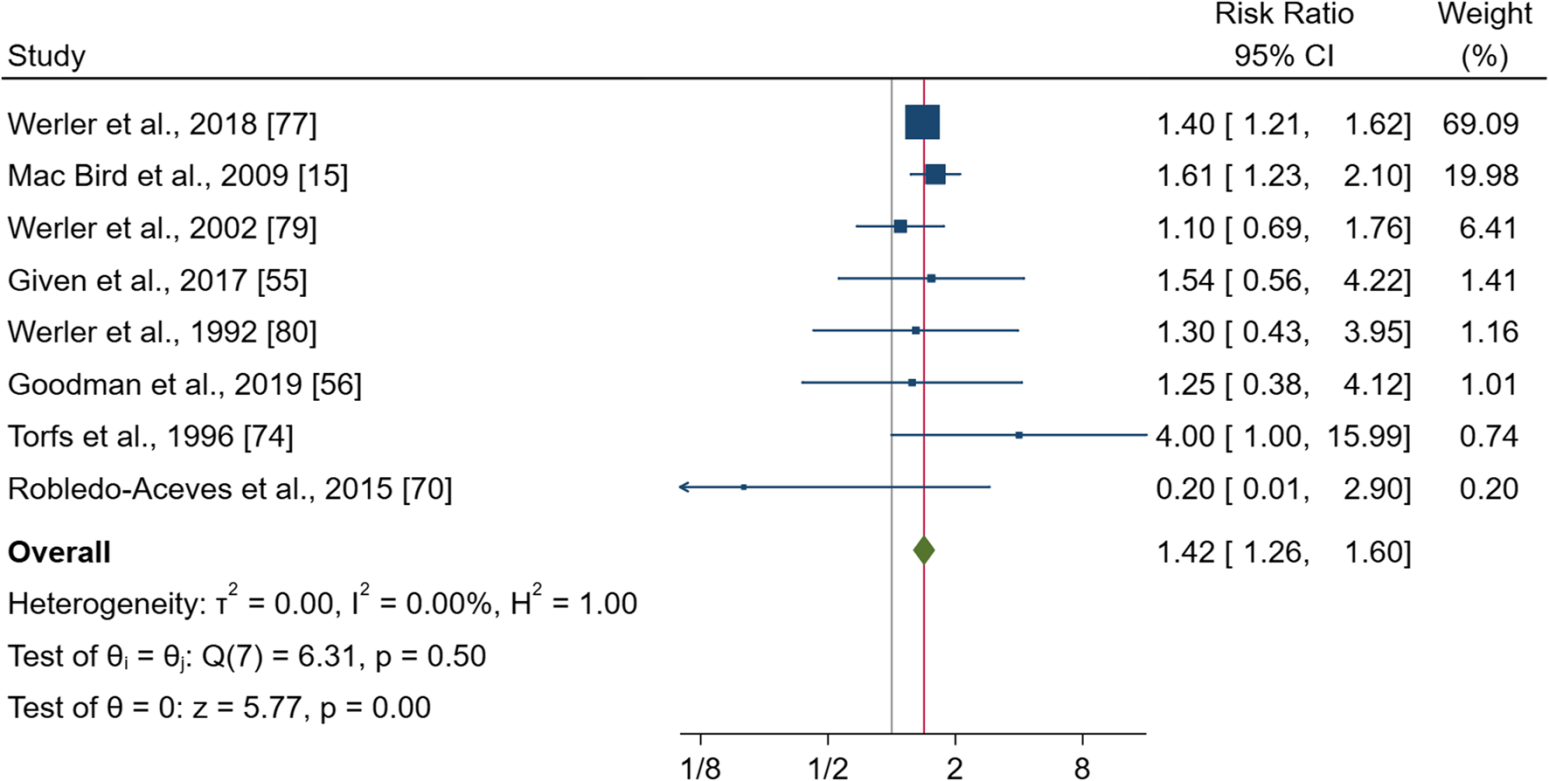
Forest plot (random-effect analysis) of the association between ibuprofen use during pregnancy and the risk of gastroschisis

Eight studies, comprising 178,267 pregnancies, examined the risk of gastroschisis in women taking ibuprofen during pregnancy compared to those non-users. The pooled effect estimate showed a significant increase in the risk with RR of 1.42 (95% CI, 1.26–1.60, p < 0.000) (Fig. 3). No heterogeneity was observed (I^2^ = 0.0%; p = 0.5). Subgroup analysis showed significantly increased risk for women living in North America and taking aspirin during the periconceptional period [RR = 1.43 (95% CI 1.26–1.61), p = 0.000¸ RR = 1.44 (95% CI 1.27–1.64), p = 0.000]; and for the subgroups of fully adjusted studies [RR = 1.42 (95% CI 1.25–1.60), p = 0.000], and in those studies where the control group included healthy newborns (see Additional file 1: Fig. S8). The sensitivity analysis confirmed a significantly increased risk of gastroschisis. The funnel plot showed no visual asymmetry (Additional file 1: Fig. S8a), and no publication bias was observed (Egger’s test p = 0.78).

### Decongestants

**Fig. 4:**
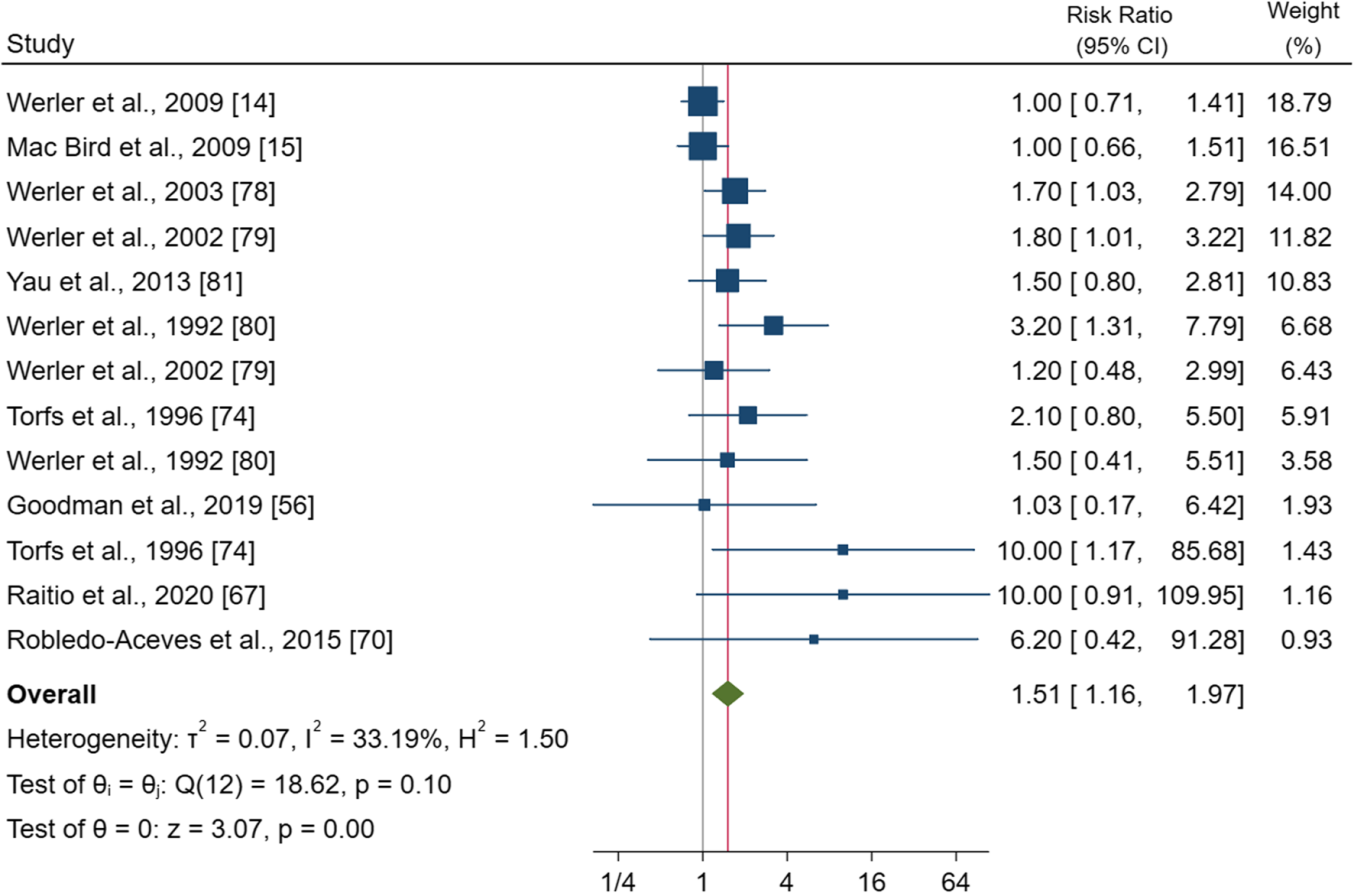
Forest plot (random-effect analysis) of the association between decongestant use during pregnancy and the risk of gastroschisis

Ten studies comprising 25,761 pregnancies were included in this analysis. The pooled effect estimate showed that the use of pseudoephedrine and phenylpropanolamine during pregnancy significantly increased the RR by 1.51 (95% CI 1.16–1.97, p = 0.00) (Fig. 4). No heterogeneity was observed (I^2^ = 33.2%; p = 0.10). Subgroup analysis showed a significantly higher risk for women living in North America [RR = 1.44 (95% CI 1.12–1.87), p = 0.005] and for those taking these two decongestants during the first trimester [RR = 1.83 (95% CI 1.41–2.39), p = 0.000]. Also, for the subgroups of fully adjusted [RR = 1.36 (95% CI 1.06–1.76), p = 0.017] or adjusted for maternal age plus lifestyle factors studies, and those where the control group were newborns with other congenital anomalies [RR = 2.51 (95% CI 1.21–5.24), p = 0.001] or newborns without anomalies plus malformed infants [RR = 1.65 (95% CI 2.16–2.34), p = 0.005] significant increases were observed (Additional file 1: Fig. S9). The sensitivity analysis showed a significantly increased risk except for NOS ≥ 7.

The funnel plot showed a right-hand side asymmetry (Additional file 1: Fig. S9a) confirmed by Egger’s test (p = 0.002). The trim-and-fill procedure imputed 3 studies and suggested a correct RR of 1.41 (95% CI 1.09–1.81).

### Paracetamol

**Fig. 5:**
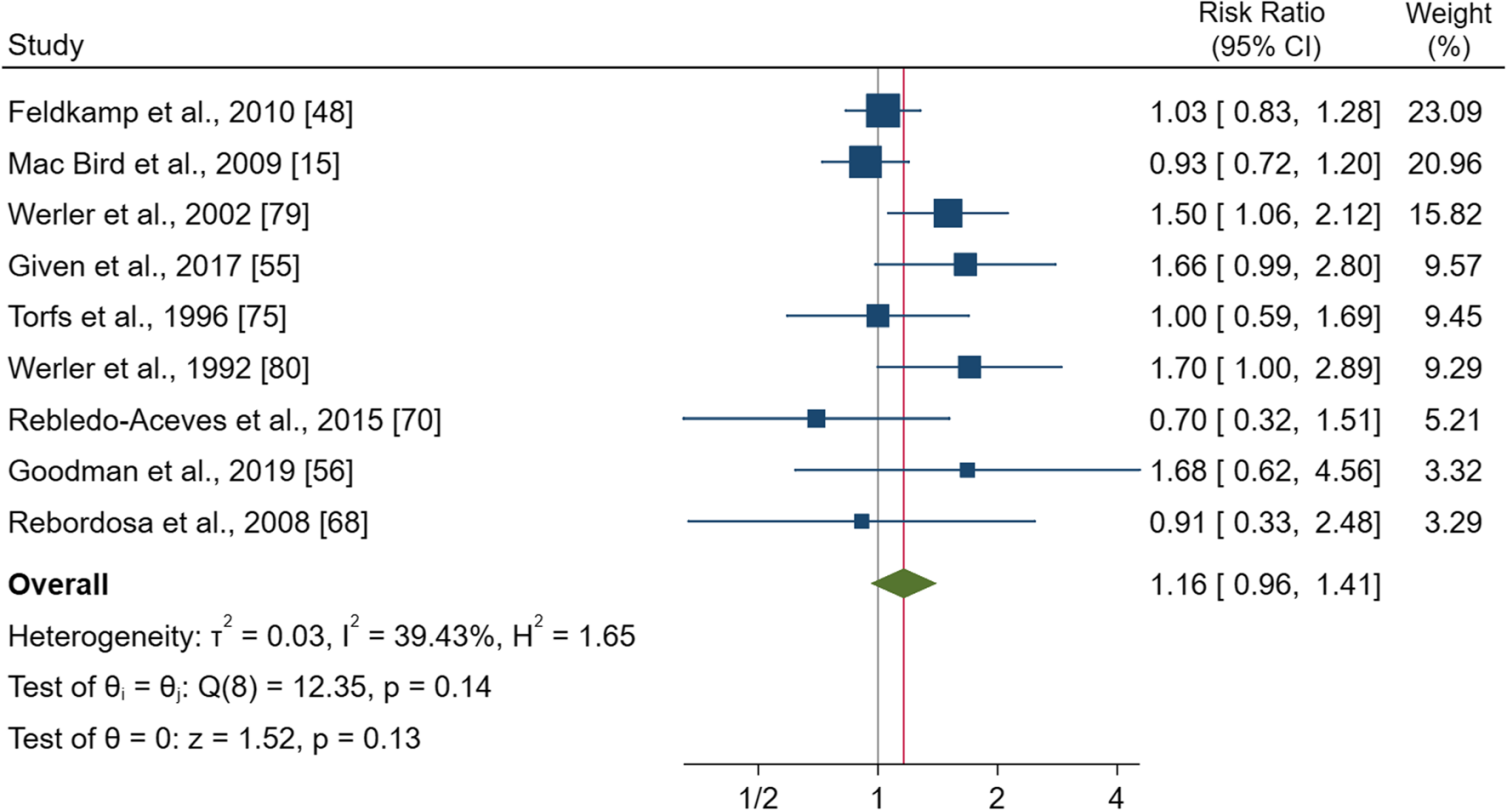
Forest plot (random-effect analysis) of the association between paracetamol use during pregnancy and the risk of gastroschisis

Nine studies comprising 190,483 pregnancies examined the risk of gastroschisis in women taking paracetamol compared to those non-users. The pooled effect estimate showed no significantly increased risk of gastroschisis with a RR of 1.16 (95% CI 0.96–1.41), p = 0.13) (Fig. 5). No heterogeneity was observed (I^2^ = 39.4%; p = 0.14). The subgroup analysis showed significant increases for the subgroups of studies where the control group were newborns without congenital anomalies (Additional file 1: Fig. S10).

The funnel plot showed no visual asymmetry (Additional file 1: Fig. S10a), and no publication bias was observed (Egger’s test p = 0.66).

### Oral contraceptives

**Fig. 6:**
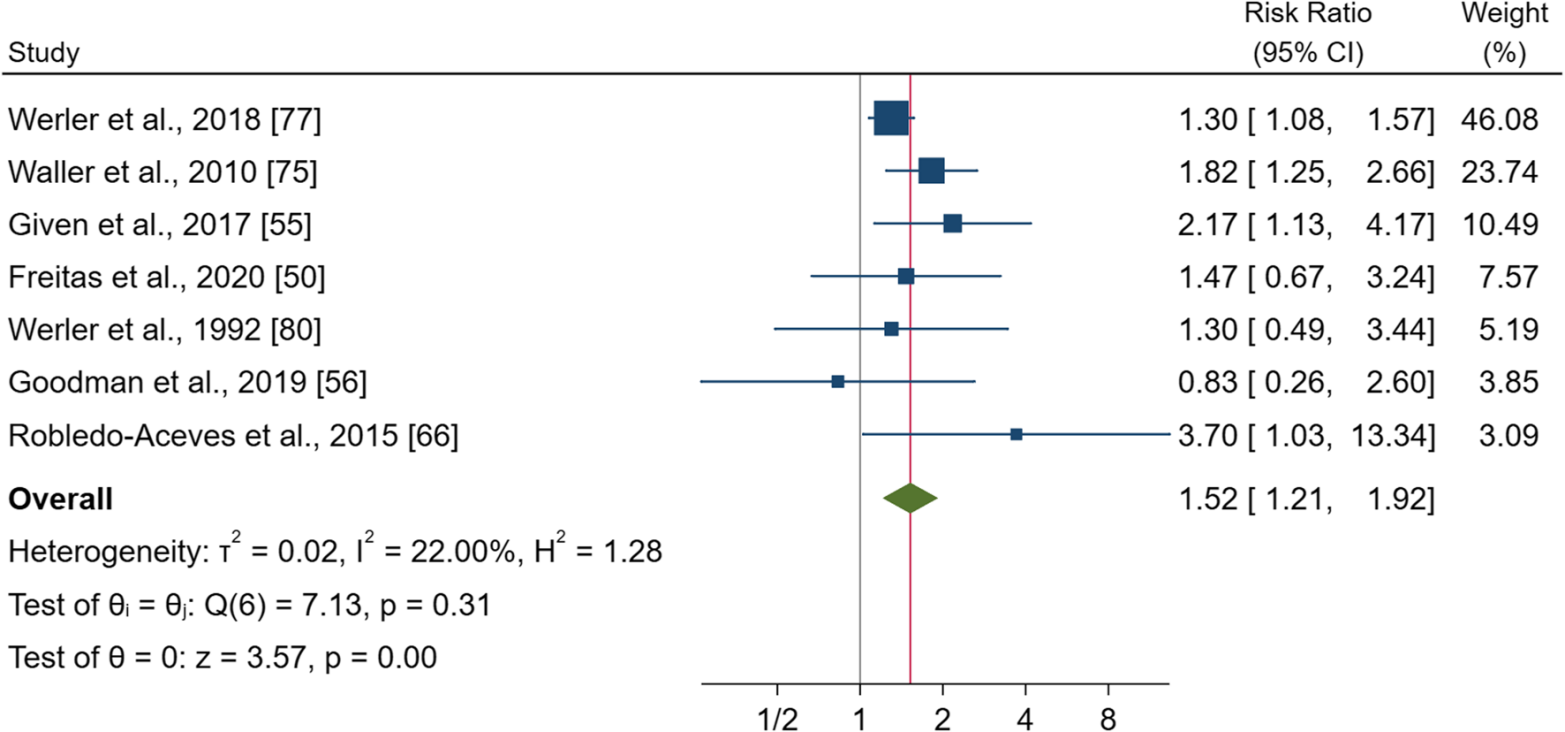
Forest plot (random-effect analysis) of the association between oral contraceptive use during pregnancy and the risk of gastroschisis

Seven studies comprising 176,086 pregnancies were included in this analysis. The pooled effect estimate showed that gestational use was associated with a significantly increased risk of gastroschisis with a RR of 1.52 (95% CI 1.21–1.92, p < 0.000) (Fig. 6). No heterogeneity was observed (I^2^ = 22.0%; p = 0.31). Subgroups analysis showed significantly increased risk for women living in North America [RR = 1.40 (95% CI 1.10–1.79), p = 0.006]; (also, in Europe but only in 1 study) and for all other subgroups (Additional file 1: Fig. S11). The sensitivity analysis confirmed significant increases in the risk of gastroschisis.

The funnel plot showed no visual asymmetry (Additional file 1: Fig. S11a), and no publication bias was observed (Egger’s test p = 0.44).

## Discussion

We conducted meta-analyses of 18 studies from 28 countries, including 751,954 pregnancies spanning 25 years. These meta-analyses suggested that users in the first trimester of pregnancy of over the counter medications (OTC) such as aspirin, ibuprofen, pseudoephedrine and phenylpropanolamine, and oral contraceptives, were associated with an increased risk of gastroschisis in offspring from 1.4 to 1.6 times greater than no users, at the 95% confidence.

Conversely, for paracetamol gestational use, no significant association was observed.

To the best of our knowledge, this is the first systematic review and meta-analysis that provides an overview of the available epidemiological studies examining the association between gestational medication use and the risk of gastroschisis.

These findings are according to the previous meta-analysis by Kozer et al. [19] focused on aspirin use only, that included 5 studies published until 2000, and showed a significant increased risk of gastroschisis for aspirin users during the first trimester.

Several studies have shown that medications use during pregnancy has a teratogenic effect on humans and suggested that oxidative stress is one of the main teratogenic mechanism involved in a wide spectrum of congenital anomalies, foetal growth retardation and in severe cases of *in-utero* death [84]. In particular, human evidence has consistently showed that the presence of oxidative stress biomarkers may lead to inflammation or might affect the placenta during the early stage of organogenesis demonstrated the relationship between unbalanced oxidative level and the occurrence of adverse pregnancy outcomes [85].

These findings are consistent with Bargy and Beaudoin [2] and Beaudoin [7] embryo researches which showed that the pathogenetic mechanism of gastroschisis could be due to teratogenic agents and that the rapture of the amnion predominantly occurs at 8 weeks.

Other epidemiological findings showed that infections acquired during the first trimester of pregnancy, are associated with gastroschisis, likely through immune and inflammatory pathway [86–89].

However, since these OTC medications are used for common illnesses, such as maternal fever and upper respiratory infection, questions have been raised about interactions between medications and potential confounding by an underlying illness.

Paracetamol is one of the most widely used OTC analgesic and antipyretic medications. Our findings are consistently with the review of Wang et al. 2017. Several in vivo and in vitro studies showed that paracetamol is safe when used at therapeutic dose and only a paracetamol overdose can cause oxidative stress [90].

The relationship between oestrogen and oxidative stress activation was proposed by Lubinsky et al. [91]. Several studies suggested an increased oxidative stress in combined oestrogens and progestin users [92–99] with very high hormone levels were detected among healthy young women [98, 99].

However, these findings indicate that the physicians should closely manage medications therapy during pregnancy to optimize the therapeutic regimens at the individual level [100].

Subgroups analysis shows a specific population-related effect as gestational use of aspirin and oral contraceptives report a significant increase in the risk of gastroschisis only in North America. These geographic variations may reflect country-specific maternal lifestyle habits as well as specific sociodemographic characteristics [20]. Furthermore, the results of subgroups analysis indicate that for aspirin and decongestants, the increases are significant only during first-trimester exposure, the critical period for gastroschisis development [7]. For ibuprofen, the exposure during the periconceptional time determines a significant increase, while, for oral contraceptives, the increase was observed both in periconceptional and first-trimester exposure time. Additionally, for all individual medications, only those studies with fully adjustment variables report significant increases in risk. Still, for contraceptives, it was also observed when the study was adjusted for maternal age only. It is important to note that the highest pooled RR for aspirin is reported by only 2 studies adjusted for maternal age and lifestyle risk factors associated with an increased risk for gastroschisis, as observed in a previous study [20].

Regarding the type of control, the subgroup analysis for oral contraceptives shows significant increases when cases are compared to healthy newborns. For the two individual decongestants, the increase is significant when malformed and both healthy and malformed newborns are considered as controls. However, an under or an overestimation of the exposure among the mothers of healthy or unhealthy newborns cannot be ruled out.

Publication bias may have affected aspirin and decongestants' findings, resulting in an overestimation of the statistical significance of the results. However, the trim-and-filled procedure was imputed at few potentially unpublished studies (2 for aspirin; 3 for decongestants), providing a correct RR that confirmed the presence of association. Moreover, as multiple comparisons were carried out, an overestimation of statistical results cannot be excluded.

Our study has several strengths. These meta-analyses included many large, multicenter, population-based studies that allow ample statistical power. In most of the studies, gastroschisis cases were ascertained by rigorous birth defect surveillance methods, including live births, stillbirths and terminations of pregnancy, which reduce potential misclassification due to incomplete ascertainment. Additionally, most of all included studies were adjusted for several confounders reducing biased for residual confounders. Moreover, sensitivity analysis suggested that our results were not influenced by heterogeneity across the studies.

However, several limitations also must be considered. First, included case–control studies may be affected by selection and recall bias. Second, since OTC medications do not required a medical prescription, is very difficult to obtain accurate data on pregnancy exposure due to the absence of pharmacy documentation or medical records. Therefore, when studies relied on self-reported and retrospective exposure assessment, the results might be affected by exposure misclassification and recall bias, particularly for medications like individual non-steroidal anti-inflammatory drugs (NSAIDS), usually used for short-term treatments of common illnesses. Third, these observational studies did not evaluate the dose and/or the frequency of medication use, as well as their combined use and, thereby, their possible interactions. Fourth, despite most studies adjusted for several potential confounders, we acknowledge that the residual confounding by unmeasured factors remains possible. Nevertheless, adjusting for potential confounders, including interactions between medications, is necessary. Moreover, some studies have a small sample size. Consequently, the power to detect an association is low. Finally, our review was limited to English language publications, even if non-English language articles are not all available on PubMed, Scopus and EMBASE databases.

## Conclusions

Meta-analysis results suggested that OTC medications such as aspirin, ibuprofen, pseudoephedrine, phenylpropanolamine, as well as oral contraceptives during the first trimester of pregnancy are associated with a moderate but significantly increased risk of gastroschisis. However, these associations are significant only in particular subgroups defined by geographic location, adjustment variables and type of control. Due to the absence of the dosage and frequency of medication use, care should be taken when drawing general conclusions. Moreover, in pharmacoepidemiology research, the distinction between the statistical significance and the clinical meaning must always be considered. Further studies, with large sample size and well-planned methodology, including a dose–response effect, are warranted to verify these findings and to assess individual medication safety to help clinicians decide on their prescription during early pregnancy.

### Supplementary Information

**Additional file 1: Table S1.** Details of search strategy.** Table S2. A** Newcastle-Ottawa Scale quality assessment - case-control studies.** B** Newcastle-Ottawa Scale quality assessment - cohort studies.** Table S3.** Summary of studies excluded from the meta-analysis on gestational medication use and risk for gastroschisis (listed alphabetically by the first author).** Table S4.** Newcastle-Ottawa Scale quality assessment of the studies included in meta-analysis.

## Data Availability

Not applicable.

## Abbreviations

ATC: Anatomical Therapeutic Chemical
CI: Confidence interval
HR: Hazard ratio
NOS: Newcastle–Ottawa scale
NSAIDS: Non-steroidal anti-inflammatory drugs
OR: Odds ratio
OTC: Over the counter medications
PRISMA: Preferred Reporting Items for Systematic reviews and Meta-analyses
RR: Risk ratio
USA: United states of America

## References

[1] Rittler M, Vauthay L, Mazzitelli N (2013). Gastroschisis is a defect of the umbilical ring: evidence from morphological evaluation of stillborn fetuses. Birth Defects Res A Clin Mol Teratol.

[2] Bargy F, Beaudoin S (2014). Comprehensive developmental mechanisms in gastroschisis. Fetal Diagn Ther.

[3] Mastroiacovo P, Lisi A, Castilla EE, Martínez-Frías ML, Bermejo E, Marengo L (2007). Gastroschisis and associated defects: an international study. Am J Med Genet A.

[4] Youssef F, Cheong LH, Emil S, Canadian Pediatric Surgery Network (CAPSNet) (2016). Gastroschisis outcomes in North America: a comparison of Canada and the United States. J Pediatr Surg.

[5] Vo LU, Langlois PH (2015). Time trends in prevalence of gastroschisis in Texas, 1999 to 2011: subgroup analyses by maternal and infant characteristics. Birth Defects Res A Clin Mol Teratol.

[6] Lepigeon K, Van Mieghem T, Vasseur Maurer S, Giannoni E, Baud D (2014). Gastroschisis-what should be told to parents?. Prenat Diagn.

[7] Beaudoin S (2018). Insights into the etiology and embryology of gastroschisis. Semin Pediatr Surg.

[8] Jones AM, Isenburg J, Salemi JL, Arnold KE, Mai CT, Aggarwal D, Arias W, Carrino GE, Ferrell E, Folorunso O, Ibe B, Kirby RS, Krapfl HR, Marengo LK, Mosley BS, Nance AE, Romitti PA, Spadafino J, Stock J, Honein MA (2016). Increasing prevalence of gastroschisis–14 states, 1995–2012. MMWR Morb Mortal Wkly Rep.

[9] Loane M, Dolk H, Bradbury I, EUROCAT Working Group (2007). Increasing prevalence of gastroschisis in Europe 1980–2002: a phenomenon restricted to younger mothers?. Paediatr Perinat Epidemiol.

[10] Castilla EE, Mastroiacovo P, Orioli IM (2008). Gastroschisis: international epidemiology and public health perspectives. Am J Med Genet C Semin Med Genet.

[11] Rittler M, Campaña H, Ermini ML, Gili JA, Poletta FA, Pawluk MS (2015). Gastroschisis and young mothers: What makes them different from other mothers of the same age?. Birth Defects Res A Clin Mol Teratol.

[12] Skarsgard ED, Meaney C, Bassil K, Brindle M, Arbour L, Moineddin R, Canadian Pediatric Surgery Network (CAPSNet) (2015). Maternal risk factors for gastroschisis in Canada. Birth Defects Res A Clin Mol Teratol.

[13] Feldkamp ML, Carey JC, Sadler TW (2007). Development of gastroschisis: review of hypotheses, a novel hypothesis, and implications for research. Am J Med Genet A.

[14] Werler MM, Mitchell AA, Moore CA, Honein MA, National Birth Defects Prevention Study (2009). Is there epidemiologic evidence to support vascular disruption as a pathogenesis of gastroschisis?. Am J Med Genet A.

[15] Mac Bird T, Robbins JM, Druschel C, Cleves MA, Yang S, Hobbs CA (2009). Demographic and environmental risk factors for gastroschisis and omphalocele in the National Birth Defects Prevention Study. J Pediatr Surg.

[16] Curry JI, McKinney P, Thornton JG, Stringer MD (2000). The aetiology of gastroschisis. BJOG.

[17] Rasmussen SA, Frías JL (2008). Non-genetic risk factors for gastroschisis. Am J Med Genet C Semin Med Genet.

[18] Frolov P, Alali J, Klein MD (2010). Clinical risk factors for gastroschisis and omphalocele in humans: a review of the literature. Pediatr Surg Int.

[19] Kozer E, Nikfar S, Costei A, Boskovic R, Nulman I, Koren G (2002). Aspirin consumption during the first trimester of pregnancy and congenital anomalies: a meta-analysis. Am J Obstet Gynecol.

[20] Baldacci S, Santoro M, Coi A, Mezzasalma L, Bianchi F, Pierini A (2020). Lifestyle and sociodemographic risk factors for gastroschisis: a systematic review and meta-analysis. Arch Dis Child.

[21] Page MJ, Moher D, Bossuyt PM, Boutron I, Hoffmann TC, Mulrow CD (2021). PRISMA 2020 explanation and elaboration: updated guidance and exemplars for reporting systematic reviews. BMJ.

[22] Wells GA, Shea B, O'Connell D, Peterson J, Welch V, Losos M, et al. The Newcastle-Ottawa Scale (NOS) for assessing the quality of nonrandomised studies in meta-analyses. Ottawa Hospital Research Institute. Ottawa Scale [Internet]. 2020 Available from: https://www.ohri.ca/programs/clinical_epidemiology/oxford.asp.

[23] Xie P, Xia W, Lowe S, Zhou Z, Ding P, Cheng C, Bentley R, Li Y, Wang Y, Zhou Q, Wu B, Gao J, Feng L, Ma S, Liu H, Sun C (2022). High spicy food intake may increase the risk of esophageal cancer: a meta-analysis and systematic review. Nutr Res.

[24] Gorelik E, Masarwa R, Perlman A, Rotshild V, Abbasi M, Muszkat M, Matok I (2019). Fluoroquinolones and cardiovascular risk: a systematic review, meta-analysis and network meta-analysis. Drug Saf.

[25] Ma Z, Cao X, Chang Y, Li W, Chen X, Tang NJ (2021). Association between gestational exposure and risk of congenital heart disease: a systematic review and meta-analysis. Environ Res.

[26] Fell M, Dack K, Chummun S, Sandy J, Wren Y, Lewis S (2022). Maternal cigarette smoking and cleft lip and palate: a systematic review and meta-analysis. Cleft Palate Craniofac J.

[27] Wang Z, Brauer R, Man KKC, Alfageh B, Mongkhon P, Wong ICK (2021). Prenatal exposure to antipsychotic agents and the risk of congenital malformations in children: a systematic review and meta-analysis. Br J Clin Pharmacol.

[28] Li P, Qin X, Tao F, Huang K (2020). Maternal exposure to sulfonamides and adverse pregnancy outcomes: a systematic review and meta-analysis. PLoS ONE.

[29] Higgins JP, Thompson SG (2002). Quantifying heterogeneity in a meta-analysis. Stat Med.

[30] Egger M, Davey Smith G, Schneider M, Minder C (1997). Bias in meta­analysis detected by a simple, graphical test. BMJ.

[31] Sterne JA, Gavaghan D, Egger M (2000). Publication and related bias in meta-analysis: power of statistical tests and prevalence in the literature. J Clin Epidemiol.

[32] Duval S, Tweedie R (2000). A nonparametric, “trim and fill” method of accounting for publication bias in meta-analysis. J Am Stat Assoc.

[33] Murad MH, Chu H, Lin L, Wang Z (2018). The effect of publication bias magnitude and direction on the certainty in evidence. BMJ Evid Based Med.

[34] Ahrens KA, Anderka MT, Feldkamp ML, Canfield MA, Mitchell AA, Werler MM (2013). Antiherpetic medication use and the risk of gastroschisis: findings from the National Birth Defects Prevention Study, 1997–2007. Paediatr Perinat Epidemiol.

[35] Ailes EC, Gilboa SM, Gill SK, Broussard CS, Crider KS, Berry RJ (2016). Association between antibiotic use among pregnant women with urinary tract infections in the first trimester and birth defects, National Birth Defects Prevention Study 1997 to 2011. Birth Defects Res A Clin Mol Teratol.

[36] Alwan S, Reefhuis J, Rasmussen SA, Olney RS, Friedman JM (2007). Use of selective serotonin-reuptake inhibitors in pregnancy and the risk of birth defects. N Engl J Med.

[37] Anderson KN, Dutton AC, Broussard CS, Farr SL, Lind JN, Visser SN (2020). ADHD medication use during pregnancy and risk for selected birth defects: national birth defects prevention study, 1998–2011. J Atten Disord.

[38] Anderson KN, Ailes EC, Lind JN, Broussard CS, Bitsko RH, Friedman JM (2020). Atypical antipsychotic use during pregnancy and birth defect risk: National Birth Defects Prevention Study, 1997–2011. Schizophr Res.

[39] Bitsko RH, Reefhuis J, Louik C, Werler M, Feldkamp ML, Waller DK (2008). Periconceptional use of weight loss products including ephedra and the association with birth defects. Birth Defects Res A Clin Mol Teratol.

[40] Blotière PO, Raguideau F, Weill A, Elefant E, Perthus I, Goulet V (2019). Risks of 23 specific malformations associated with prenatal exposure to 10 antiepileptic drugs. Neurology.

[41] Broussard CS, Rasmussen SA, Reefhuis J, Friedman JM, Jann MW, Riehle-Colarusso T (2011). Maternal treatment with opioid analgesics and risk for birth defects. Am J Obstet Gynecol.

[42] Carter TC, Druschel CM, Romitti PA, Bell EM, Werler MM, Mitchell AA (2008). Antifungal drugs and the risk of selected birth defects. Am J Obstet Gynecol.

[43] Charlton BM, Mølgaard-Nielsen D, Svanström H, Wohlfahrt J, Pasternak B, Melbye M (2016). Maternal use of oral contraceptives and risk of birth defects in Denmark: prospective, nationwide cohort study. BMJ.

[44] Crider KS, Cleves MA, Reefhuis J, Berry RJ, Hobbs CA, Hu DJ (2009). Antibacterial medication use during pregnancy and risk of birth defects: National Birth Defects Prevention Study. Arch Pediatr Adolesc Med.

[45] David AL, Holloway A, Thomasson L, Syngelaki A, Nicolaides K, Patel RR (2014). A case-control study of maternal periconceptual and pregnancy recreational drug use and fetal malformation using hair analysis. PLoS ONE.

[46] Draper ES, Rankin J, Tonks AM, Abrams KR, Field DJ, Clarke M, Kurinczuk JJ (2008). Recreational drug use: a major risk factor for gastroschisis?. Am J Epidemiol.

[47] Feldkamp ML, Meyer RE, Krikov S, Botto LD (2010). Acetaminophen use in pregnancy and risk of birth defects: findings from the National Birth Defects Prevention Study. Obstet Gynecol.

[48] Fisher SC, Van Zutphen AR, Werler MM, Romitti PA, Cunniff C, Browne ML (2018). Maternal antihypertensive medication use and selected birth defects in the National Birth Defects Prevention Study. Birth Defects Res.

[49] Furu K, Kieler H, Haglund B, Engeland B, Selmer A, Stephansson R (2015). Selective serotonin reuptake inhibitors and venlafaxine in early pregnancy and risk of birth defects: population based cohort study and sibling design. BMJ.

[50] Garne E, Hansen AV, Morris J, Zaupper L, Addor MC, Barisic I (2015). Use of asthma medication during pregnancy and risk of specific congenital anomalies: a European case-malformed control study. J Allergy Clin Immunol.

[51] van Gelder MM, Van Bennekom CM, Louik C, Werler MM, Roeleveld N, Mitchell AA (2015). Maternal hypertensive disorders, antihypertensive medication use, and the risk of birth defects: a case–control study. BJOG.

[52] Gilboa SM, Strickland MJ, Olshan AF, Werler MM, Correa A (2009). Use of antihistamine medications during early pregnancy and isolated major malformations. Birth Defects Res A Clin Mol Teratol.

[53] Howley MM, Papadopoulos EA, Van Bennekom CM, Van Zutphen AR, Carmichael SL, Munsie JW (2020). Asthma medication use and risk of birth defects: National Birth Defects Prevention Study, 1997–2011. J Allergy Clin Immunol Pract.

[54] Interrante JD, Ailes EC, Lind JN, Anderka M, Feldkamp ML, Werler MM (2017). Risk comparison for prenatal use of analgesics and selected birth defects, National Birth Defects Prevention Study 1997–2011. Ann Epidemiol.

[55] Jenkins MM, Reefhuis J, Gallagher ML, Mulle JG, Hoffmann TJ, Koontz DA (2014). Maternal smoking, xenobiotic metabolizing enzyme gene variants, and gastroschisis risk. Am J Med Genet A.

[56] Lam PK, Torfs CP, Brand RJ (1999). A low pregnancy body mass index is a risk factor for an offspring with gastroschisis. Epidemiology.

[57] Li Q, Mitchell AA, Werler MM, Yau WP, Hernández-Díaz S (2013). Assessment of antihistamine use in early pregnancy and birth defects. J Allergy Clin Immunol Pract.

[58] Lin S, Munsie JP, Herdt-Losavio ML, Bell E, Druschel C, Romitti PA (2008). Maternal asthma medication use and the risk of gastroschisis. Am J Epidemiol.

[59] Louik C, Ahrens K, Kerr S, Pyo J, Chambers C, Jones KL (2013). Risks and safety of pandemic H1N1 influenza vaccine in pregnancy: exposure prevalence, preterm delivery, and specific birth defects. Vaccine.

[60] Paranjothy S, Broughton H, Evans A, Huddart S, Drayton M, Jefferson R (2012). The role of maternal nutrition in the aetiology of gastroschisis: an incident case-control study. Int J Epidemiol.

[61] Polen KN, Rasmussen SA, Riehle-Colarusso T, Reefhuis J (2013). Association between reported venlafaxine use in early pregnancy and birth defects, national birth defects prevention study, 1997–2007. Birth Defects Res A Clin Mol Teratol.

[62] Reefhuis J, Devine O, Friedman JM, Louik C, Honein MA (2015). Specific SSRIs and birth defects: Bayesian analysis to interpret new data in the context of previous reports. BMJ.

[63] Siega-Riz AM, Herring AH, Olshan AF, Smith J, Moore C (2008). The joint effects of maternal prepregnancy body mass index and age on the risk of gastroschisis. Paediatr Perinat Epidemiol.

[64] Tinker SC, Reefhuis J, Bitsko RH, Gilboa SM, Mitchell AA, Tran EL (2019). Use of benzodiazepine medications during pregnancy and potential risk for birth defects, National Birth Defects Prevention Study, 1997–2011. Birth Defects Res.

[65] Torfs CP, Lam PK, Schaffer DM, Brand RJ (1998). Association between mothers' nutrient intake and their offspring's risk of gastroschisis. Teratology.

[66] Wemakor A, Casson K, Garne E, Bakker M, Addor MC, Arriola L (2015). Selective serotonin reuptake inhibitor antidepressant use in first trimester pregnancy and risk of specific congenital anomalies: a European register-based study. Eur J Epidemiol.

[67] Feldkamp ML, Carmichael SL, Shaw GM, Panichello JD, Moore CA, Botto LD (2011). Maternal nutrition and gastroschisis: findings from the National Birth Defects Prevention Study. Am J Obstet Gynecol.

[68] Freitas AB, Centofanti SF, Osmundo-Junior GS, Rodrigues AS, Francisco RPV, Brizot ML (2020). Risk factors for gastroschisis: a case–control study in a Brazilian population. Int J Gynecol Obstet.

[69] Given JE, Loane M, Garne E, Nelen V, Barisic I, Randrianaivo H (2017). Gastroschisis in Europe: a case-malformed–control study of medication and maternal illness during pregnancy as risk factors. Paediatr Perinat Epidemiol.

[70] Goodman JR, Peck JD, Landmann A, Williams M, Elimian A (2019). An evaluation of nutritional and vasoactive stimulants as risk factors for gastroschisis: a pilot study. J Matern Fetal Neonatal Med.

[71] Martínez-Frías ML, Rodríguez-Pinilla E, Prieto L (1997). Prenatal exposure to salicylates and gastroschisis: a case–control study. Teratology.

[72] Raitio A, Tauriainen A, Leinonen MK, Syvänen J, Kemppainen T, Löyttyniemi E (2020). Maternal risk factors for gastroschisis: a population-based case-control study. Birth Defects Res.

[73] Rebordosa C, Kogevinas M, Horváth-Puhó E, Nørgård B, Morales M, Czeizel AE (2008). Acetaminophen use during pregnancy: effects on risk for congenital abnormalities. Am J Obstet Gynecol.

[74] Robledo-Aceves M, Bobadilla-Morales L, Mellín-Sánchez EL, Corona-Rivera A, Pérez-Molina JJ, Cárdenas-Ruiz Velasco JJ (2015). Prevalence and risk factors for gastroschisis in a public hospital from west México. Congenit Anom (Kyoto).

[75] Torfs CP, Katz EA, Bateson TF, Lam PK, Curry CJ (1996). Maternal medications and environmental exposures as risk factors for gastroschisis. Teratology.

[76] Waller DK, Gallaway MS, Taylor LG, Ramadhani TA, Canfield MA, Scheuerle A (2010). Use of oral contraceptives in pregnancy and major structural birth defects in offspring. Epidemiology.

[77] Werler MM, Guéry E, Waller DK, Parker SE (2018). Gastroschisis and cumulative stressor exposures. Epidemiology.

[78] Werler MM, Sheehan JE, Mitchell AA (2003). Association of vasoconstrictive exposures with risks of gastroschisis and small intestinal atresia. Epidemiology.

[79] Werler MM, Sheehan JE, Mitchell AA (2002). Maternal medication use and risks of gastroschisis and small intestinal atresia. Am J Epidemiol.

[80] Werler MM, Mitchell AA, Shapiro S (1992). First trimester maternal medication use in relation to gastroschisis. Teratology.

[81] Yau WP, Mitchell AA, Lin KJ, Werler MM, Hernández-Díaz S (2013). Use of decongestants during pregnancy and the risk of birth defects. Am J Epidemiol.

[82] Slone Epidemiology Center at Boston University. The Slone Drug Dictionary. https://www.bu.edu/slone/drug-dictionary/

[83] WHO Collaborating Centre for Drug Statistics Methodology, Guidelines for ATC classification and DDD assignment, 2021. Oslo, 2020. https://www.whocc.no/atc_ddd_index_and_guidelines/guidelines/

[84] van Gelder MM, van Rooij IA, Miller RK, Zielhuis GA, de Jong-van den Berg LT, Roeleveld N (2010). Teratogenic mechanisms of medical drugs. Hum Reprod Update.

[85] Toboła-Wróbel K, Pietryga M, Dydowicz P, Napierała M, Brązert J, Florek E (2020). Association of oxidative stress on pregnancy. Oxid Med Cell Longev.

[86] Feldkamp ML, Enioutina EY, Botto LD, Krikov S, Byrne JL, Geisler WM (2015). Chlamydia trachomatis IgG3 seropositivity is associated with gastroschisis. J Perinatol.

[87] Feldkamp ML, Arnold KE, Krikov S, Reefhuis J, Almli LM, Moore CA (2019). Risk of gastroschisis with maternal genitourinary infections the US National birth defects prevention study 1997–2011. BMJ Open.

[88] Yazdy MM, Mitchell AA, Werler MM (2014). Maternal genitourinary infections and the risk of gastroschisis. Am J Epidemiol.

[89] Ahrens KA, Anderka MT, Feldkamp ML, Canfield MA, Mitchell AA, Werler MM, National Birth Defects Prevention Study (2013). Antiherpetic medication use and the risk of gastroschisis: findings from the National Birth Defects Prevention Study, 1997–2007. Paediatr Perinat Epidemiol.

[90] Wang X, Wu Q, Liu A, Anadón A, Rodríguez JL, Martínez-Larrañaga MR, Yuan Z, Martínez MA (2017). Paracetamol: overdose-induced oxidative stress toxicity, metabolism, and protective effects of various compounds in vivo and in vitro. Drug Metab Rev.

[91] Lubinsky M (2012). Hypothesis: estrogen-related thrombosis explains the pathogenesis and epidemiology of gastroschisis. Am J Med Genet A.

[92] Chen JT, Kotani K (2012). Oral contraceptive therapy increases oxidative stress in pre-menopausal women. Int J Prev Med.

[93] Chen JT, Kotani K (2018). Different effects of oral contraceptive and dydrogesterone treatment on oxidative stress levels in premenopausal women. J Clin Med Res.

[94] De Groote D, Perrier d’Hauterive S, Pintiaux A, Balteau B, Gerday C, Claesen J, Foidart JM (2009). Effects of oral contraception with ethinylestradiol and drospirenone on oxidative stress in women 18–35 years old. Contraception.

[95] Finco A, Belcaro G, Cesarone MR (2011). Assessment of the activity of an oral contraceptive on the levels of oxidative stress and changes in oxidative stress after co-treatment with two different types of physiological modulators with antioxidant action. Contraception.

[96] Pincemail J, Vanbelle S, Gaspard U, Collette G, Haleng J, Cheramy-Bien JP, Charlier C, Chapelle JP, Giet D, Albert A (2007). Effect of different contraceptive methods on the oxidative stress status in women aged 40 48 years from the ELAN study in the province of Liege, Belgium. Hum Reprod.

[97] Cauci S, Di Santolo M, Culhane JF, Stel G, Gonano F, Guaschino S (2008). Effects of third-generation oral contraceptives on high-sensitivity C-reactive protein and homocysteine in young women. Obstet Gynecol.

[98] Cauci S, Buligan C, Marangone M (2016). Oxidative stress in female athletes using combined oral contraceptives. Sports Med Open.

[99] Cauci S, Xodo S, Buligan C, Colaninno C, Barbina M, Barbina G, Francescato MP (2021). Oxidative stress is increased in combined oral contraceptives users and is positively associated with high-sensitivity C-reactive protein. Molecules.

[100] Holford NH, Buclin T (2012). Safe and effective variability: a criterion for dose individualization. Ther Drug Monit.

