# Supplementary material for: Medication use during pregnancy and the risk of gastroschisis: a systematic review and meta-analysis of observational studies
Source: Orphanet J Rare Dis. 2024 Jan 30;19:31. doi: 10.1186/s13023-023-02992-z (PMC10826191; doi:10.1186/s13023-023-02992-z)
Supplement: Supplementary file 1 — Additional file 1: Table S1. Details of search strategy. Table S2. A Newcastle-Ottawa Scale quality assessment - case-control studies. B Newcastle-Ottawa Scale quality assessment - cohort studies. Table S3. Summary of studies excluded from the meta-analysis on gestational medication use and risk for gastroschisis (listed alphabetically by the first author). Table S4. Newcastle-Ottawa Scale quality assessment of the studies included in meta-analysis. [file 13023_2023_2992_MOESM1_ESM.docx]

**Medication use during pregnancy and the risk of gastroschisis: a systematic review and meta-analysis of observational studies**

Silvia Baldacci*^1^ (0000-0002-7626-1202), Michele Santoro^1^ (0000-0003-0676-3036), Lorena Mezzasalma^1^, Anna Pierini^1,2^ (0000-0003-3321-9343), Alessio Coi^1^ (0000-0002-9816-3144)

* Corresponding author: [silviab@ifc.cnr.it](mailto:silviab@ifc.cnr.it)

**^1^** Unit of Epidemiology of Rare Diseases and Congenital Anomalies, Institute of Clinical Physiology, National Research Council, Pisa, Italy

^2^ Fondazione Toscana Gabriele Monasterio, Pisa, Italy.

**Table S1** Details of search strategy

**PubMed user query:**

Search: (((maternal) AND (medication)) OR (medical drug)) AND (gastroschisis) Filters: English, from 1990/01/01 - 2020/12/31

(((("maternally"[All Fields] OR "maternities"[All Fields] OR "maternity"[All Fields] OR "mothers"[MeSH Terms] OR "mothers"[All Fields] OR "maternal"[All Fields]) AND ("medic"[All Fields] OR "medical"[All Fields] OR "medicalization"[MeSH Terms] OR "medicalization"[All Fields] OR "medicalizations"[All Fields] OR "medicalize"[All Fields] OR "medicalized"[All Fields] OR "medicalizes"[All Fields] OR "medicalizing"[All Fields] OR "medically"[All Fields] OR "medicals"[All Fields] OR "medicated"[All Fields] OR "medication s"[All Fields] OR "medics"[All Fields] OR "pharmaceutical preparations"[MeSH Terms] OR ("pharmaceutical"[All Fields] AND "preparations"[All Fields]) OR "pharmaceutical preparations"[All Fields] OR "medication"[All Fields] OR "medications"[All Fields])) OR (("medic"[All Fields] OR "medical"[All Fields] OR "medicalization"[MeSH Terms] OR "medicalization"[All Fields] OR "medicalizations"[All Fields] OR "medicalize"[All Fields] OR "medicalized"[All Fields] OR "medicalizes"[All Fields] OR "medicalizing"[All Fields] OR "medically"[All Fields] OR "medicals"[All Fields] OR "medicated"[All Fields] OR "medication s"[All Fields] OR "medics"[All Fields] OR "pharmaceutical preparations"[MeSH Terms] OR ("pharmaceutical"[All Fields] AND "preparations"[All Fields]) OR "pharmaceutical preparations"[All Fields] OR "medication"[All Fields] OR "medications"[All Fields]) AND "drug"[All Fields])) AND ("gastroschisis"[MeSH Terms] OR "gastroschisis"[All Fields] OR "gastroschises"[All Fields])) AND ((1990/01/01:2020/12/31[pdat]) AND (english[Filter]))

Translations

maternal: "maternally"[All Fields] OR "maternities"[All Fields] OR "maternity"[All Fields] OR "mothers"[MeSH Terms] OR "mothers"[All Fields] OR "maternal"[All Fields]

medication: "medic"[All Fields] OR "medical"[All Fields] OR "medicalization"[MeSH Terms] OR "medicalization"[All Fields] OR "medicalizations"[All Fields] OR "medicalize"[All Fields] OR "medicalized"[All Fields] OR "medicalizes"[All Fields] OR "medicalizing"[All Fields] OR "medically"[All Fields] OR "medicals"[All Fields] OR "medicated"[All Fields] OR "medication's"[All Fields] OR "medics"[All Fields] OR "pharmaceutical preparations"[MeSH Terms] OR ("pharmaceutical"[All Fields] AND "preparations"[All Fields]) OR "pharmaceutical preparations"[All Fields] OR "medication"[All Fields] OR "medications"[All Fields]

medical: "medic"[All Fields] OR "medical"[All Fields] OR "medicalization"[MeSH Terms] OR "medicalization"[All Fields] OR "medicalizations"[All Fields] OR "medicalize"[All Fields] OR "medicalized"[All Fields] OR "medicalizes"[All Fields] OR "medicalizing"[All Fields] OR "medically"[All Fields] OR "medicals"[All Fields] OR "medicated"[All Fields] OR "medication's"[All Fields] OR "medics"[All Fields] OR "pharmaceutical preparations"[MeSH Terms] OR ("pharmaceutical"[All Fields] AND "preparations"[All Fields]) OR "pharmaceutical preparations"[All Fields] OR "medication"[All Fields] OR "medications"[All Fields]

gastroschisis: "gastroschisis"[MeSH Terms] OR "gastroschisis"[All Fields] OR "gastroschises"[All Fields]

AND (("1990/01/01"[PDAT]: "2020/12/31"[PDAT]) AND English[lang])

**EMBASE search strategy:**

('gastroschisis'/exp OR gastroschisis) AND ((maternal AND 'drug therapy'))

AND [article]/lim

AND [english]/lim

AND [1990-2020]/py

**Scopus search strategy:**

( ALL ( maternal ) AND ALL ( medication ) OR ALL ( medical AND drug ) AND ALL ( gastroschisis ) ) AND PUBYEAR > 1989 AND PUBYEAR < 2021 AND ( LIMIT-TO ( DOCTYPE , "ar" ) ) AND ( LIMIT-TO ( LANGUAGE , "English" ) )

**Table S2 a**

***NEWCASTLE - OTTAWA QUALITY ASSESSMENT SCALE***

***CASE CONTROL STUDIES***

Note: A study can be awarded a maximum of one star for each numbered item within the Selection and Exposure categories. A maximum of two stars can be given for Comparability.

1. **Selection** (*max of 4 stars can be given*)

1) Is the case definition adequate?

☐ a) yes, with independent validation **🟑**

☐ b) yes, e.g. record linkage or based on self reports

☐ c) no description

2) Representativeness of the cases

☐ a) consecutive or obviously representative series of cases **🟑**

☐ b) potential for selection biases or not stated

3) Selection of Controls

☐ a) community controls **🟑**

☐ b) hospital controls

☐ c) no description

4) Definition of Controls

X a) no history of disease (endpoint) **🟑**

☐ b) no description of source

**Comparability** (*max of 2 stars can be given*)

1) Comparability of cases and controls on the basis of the design or analysis

☐ a) study controls for _______________ (Select the most important factor) **🟑**^§^

☐ b) study controls for any additional factor **🟑** (These criteria could be modified to indicate specific

control for a second important factor.)

**Exposure** (*max of 3 stars can be given*)

1) Ascertainment of exposure

☐ a) secure record (e.g. surgical records) **🟑**

☐ b) structured interview where blind to case/control status **🟑**

☐ c) interview not blinded to case/control status

☐ d) written self-report or medical record only

☐ e) no description

2) Same method of ascertainment for cases and controls

☐ a) yes **🟑**

☐ b) no

3) Non-Response rate

☐ a) same rate for both groups **🟑^ƒ^**

☐ b) non-respondents described

☐ c) rate different and no designation

**^§^**maternal age; **^ƒ^** a difference up to 5% was accepted.

***NEWCASTLE - OTTAWA QUALITY ASSESSMENT SCALE***

***COHORT STUDIES***

Note: A study can be awarded a maximum of one star for each numbered item within the **Selection** and **Outcome** categories. A maximum of two stars can be given for **Comparability**

1) **Selection** (*max of 4 stars can be given*)

Representativeness of the exposed cohort

☐ a) truly representative of the average _______________ (describe) in the community **🟑**

☐ b) somewhat representative of the average ______________ in the community **🟑**

☐ c) selected group of users e.g. nurses, volunteers

☐ d) no description of the derivation of the cohort

2) Selection of the non-exposed cohort

☐ a) drawn from the same community as the exposed cohort **🟑**

☐ b) drawn from a different source

☐ c) no description of the derivation of the non-exposed cohort

3) Ascertainment of exposure

☐ a) secure record (e.g. surgical records) **🟑**

☐ b) structured interview **🟑**

☐ c) written self-report

☐ d) no description

4) Demonstration that outcome of interest was not present at start of study

☐ a) yes **🟑**

☐ b) no

**Comparability** (*max of 2 stars can be given*)

1) Comparability of cohorts on the basis of the design or analysis

☐ a) study controls for _____________ (select the most important factor) **🟑^§^**

☐ b) study controls for any additional factor**🟑** (These criteria could be modified to indicate specific control for a second important factor.)

**Outcome** (*max of 3 stars can be given*)

1) Assessment of outcome

☐ a) independent blind assessment **🟑**

☐ b) record linkage **🟑**

☐ c) self-report

☐ d) no description

2) Was follow-up long enough for outcomes to occur

☐ a) yes (select an adequate follow up period for outcome of interest) **🟑**

☐ b) no

3) Adequacy of follow up of cohorts

☐ a) complete follow up - all subjects accounted for **🟑**

☐ b) subjects lost to follow up unlikely to introduce bias - small number lost - > 95%_ (select an adequate %) follow up, or description provided of those lost) **🟑**

☐ c) follow up rate <95% (select an adequate %) and no description of those lost

☐ d) no statement

**^§^**maternal age.

| **Study,**  **Year, Country** | **Study design/ time/data source/case ascertainment** | **Sample^a^**  **Size** | **Exposure** | **Exposure definition** | **Exposure**  **assessment** | **Window of Exposure** | **Measures of effect**  **(95% CI)** | **Adjusted**  **variables** | **NOS**  **score^b^** |
| --- | --- | --- | --- | --- | --- | --- | --- | --- | --- |
| Ahrens et al., 2013 ^[34]^  United States | Multicentre Case-control/1997-2007/birth defects surveillance systems, birth certificates or hospitals birth logs (NBDPS)/LB, SB, ET | 941 Gastroschisis cases;  8,339 controls | Antiherpetic | No antiherpetic use (Ref)  Antiherpetic use during early pregnancy  Antiherpetic use exclusively outside of early pregnancy | Computer assisted telephone interview | One month before to third months after conception | Antiherpetic  aOR 4.68 (1.15-19.03)  Antiherpetic use exclusively outside of early pregnancy  aOR 2.69 (0.81-8.93)  Women with genital herpes  Antiherpetic use  aOR 4.68 (1.65-13.28)  No antiherpetic use  aOR 3.00 (1.58-5.68) | Maternal age, BMI before conception | 7 |
| Ailes et al., 2016 ^[35]^  United States | Multicentre Case-control/ 1997-2011/birth defects surveillance systems,  birth certificates or hospitals birth logs (NBDPS)/LB, SB, ET | 608 cases;  231 controls  Gastroschisis cases: 45 | Penicillin, trimethoprim -sulfamethoxazole, nitrofurantoin, cephalosporins, as well as other, unknown or multiple antibiotics | Use, non-use | Computer assisted telephone interviews | One month before pregnancy to the end of the third month of pregnancy | Nitrofurantoin  aOR 0.53 (0.22-1.25)  Trimethoprim-sulfamethoxazole  aOR 0.43 (0.13-1.44) | Maternal BMI, race/  ethnicity | 7 |
| Alwan et al., 2007 ^[36]^  United States | Multicentre Case-control/ 1997-2002/birth defects surveillance systems,  birth certificates or hospitals birth logs  (NBDPS)/LB, SB, ET | ,9622 cases;  4,092 controls  Gastroschisis cases: 413 | Any Selective Serotonin-Reuptake Inhibitors  (SSRIs) | Exposed: any SSRI use  Unexposed: no SSRI use, or women who took non-SSRI antidepressants. | Computer assisted telephone interview | One month before to third months after conception | Any SSRI  aOR 1.3 (0.6-2.6)  P=0.42  Paroxetine only  aOR 2.9 (1.0-8.4) | Maternal race/ethnicity  presence or absence of maternal obesity, smoking, family income | 7 |
| Anderson et al., 2018^[37]^  United States | Multicentre Case-control/ 1998-2011/birth defects surveillance systems,  birth certificates or hospitals birth logs  (NBDPS)/LB, SB, ET | 31,213 cases;  11,382 controls  Gastroschisis cases: 1,381 | Attention-deficit/  hyperactivity disorder  (ADHD) medication | Exposed: maternal report of use of ≥1 product(s) in any dose, duration or frequency Unexposed: no ADHD use. | Computer assisted telephone interview | Three months before conception through the end of pregnancy | Any ADHD medication use  aOR 3.0 (1.2-7.4) | Maternal age | 7 |
| Anderson et al., 2020^[38]^  United States | Multicentre Case-control/ 1997-2011/birth defects surveillance systems,  birth certificates or hospitals birth logs  (NBDPS)/LB, SB, ET | 22,387 cases;  11,470 controls  Gastroschisis cases: 1,401 | Aripiprazole, azepine, clozapine, iloperidone, lurasidone, olanzapine, paliperidone, quetiapine, risperidone, ziprasidone | Use, non-use | Computer assisted telephone interview | One month before to third months after conception | Any antipsychotic  cOR 2.1 (0.6-7.3) |  | 6 |

| **Study,**  **Year, Country** | **Study design/ time/data source/case ascertainment** | **Sample^a^**  **Size** | **Exposure** | **Exposure definition** | **Exposure**  **assessment** | **Window of Exposure** | **Measures of effect**  **(95% CI)** | **Adjusted**  **variables** | **NOS**  **score^b^** |
| --- | --- | --- | --- | --- | --- | --- | --- | --- | --- |
| Bitsko et al., 2008^[39]^  United States | Multicentre Case-control/ 1998-2003/birth defects surveillance systems,  birth certificates or hospitals birth logs  (NBDPS)/LB, SB, ET | ,9672 cases;  3,324 controls  Gastroschisis cases:16 | Any weight loss products, ephedra | Exposed: any product use  Other: use of ephedra  Unexposed: women not using weight loss  products | Computer assisted telephone interviews | One month before pregnancy to the end of the third month of pregnancy | Any weight loss product  aOR 0.8 (0.4-1.7)  Other  aOR 1.2 (0.7-1.9) | BMI, multiple births, maternal education, smoking, alcohol,  caffeine use in the year before pregnancy, pregnancy intention | 7 |
| Blotière et al., 2019^[40]^  French | Cohort study/2011-2015/  French National Health Insurance database and hospital discharge database/LB, (SB and ET for spina bifida and renal agenesis only) | 8,794 cases;  1,886,825 pregnancies  Gastroschisis cases:245 | Carbamazepine, clonazepam, gabapentin, topiramate, lamotrigine, levetiracetam, pregabalin oxcarbazepine, valproic acid, phenobarbital | Use, non-use | Health Care claims reimbursed database | One month before trough two months after conception | Lamotrigine  cOR 5.2 (0.6-18.9) | Maternal age, acid folic intake, eligibility for universal health insurance, pregestational diabetes (at least 5 exposed case) | 9 |
| Broussard et al., 2011^[41]^  United States | Multicentre Case-control/ 1997-2005/birth defects surveillance systems,  birth certificates or hospitals birth logs  (NBDPS)/LB, SB, ET | 17,449 cases;  6,701 controls  Gastroschisis cases:726 | Codeine, pentazocine hydrocodone, tramadol, morphine, meperidine, oxycodone, propoxyphene, fentanyl methadone, hydromorphone | Use, non-use | Computer-assisted telephone  interview, | One months before conception through third month after conception | Opioid  aOR 1.8 (1.1-2.9)  Codeine  aOR 1.9 (0.94 -4.0)  Hydrocodone  aOR 3.3 (1.8-6.1)  Oxycodone  aOR 1.9 (0.73-5.1) | Maternal age, race/  ethnicity, education, presence or absence of prepregnancy obesity, presence or absence of periconceptional smoking, study center | 8 |
| Carter et al., 2008^[42]^  United States | Multicentre Case-control/ 1997-2003/birth defects surveillance systems,  birth certificates or hospitals birth logs  (NBDPS)/LB, SB, ET | 7,047 cases;  4,774 controls  Gastroschisis cases:455 | Antifungal drugs | Use, non-use | Computer-assisted telephone  interview, | First trimester | aOR 0.64 (0.25-1.62) | Maternal age, urinary  tract infections  in the first trimester | 8 |
| Charlton et al., 2016^[43]^  Denmark | Cohort study/1997-2011/ Danish National Patient Register, Danish Medical Birth Register/LB | 22,013 cases;  880,694 live births  Gastroschisis cases:138 | Oral Contraceptives | Never  >3 months before pregnancy onset (Ref.),  0-3 months before  pregnancy onset,  after pregnancy onset | The Danish National Prescription Register | 0-3 months before  pregnancy, and after pregnancy  onset | Never  aOR 0.29 (0.15-0.58)  0-3 months before  aOR 0.93 (0.55-1.56)  After pregnancy onset  aOR 0.95 (0.26-2.68) | Maternal age, calendar year, place of birth, county of residence,  married/living with partner, education, household income, parity, history of birth defects in a previous pregnancy, smoking, healthcare use | 9 |

| **Study,**  **Year, Country** | **Study design/ time/data source/outcome ascertainment** | **Sample^a^**  **Size** | **Exposure** | **Exposure definition** | **Exposure**  **assessment** | **Window of Exposure** | **Measures of effect**  **(95% CI)** | **Adjusted**  **variables** | **NOS**  **score^b^** |
| --- | --- | --- | --- | --- | --- | --- | --- | --- | --- |
| Crider et al., 2009^[44]^  United States | Multicentre Case-control/ 1997-2003/birth defects surveillance systems,  birth certificates or hospitals birth logs  (NBDPS)/LB, SB, ET | 13,155 cases;  4,941 controls  Gastroschisis cases:501 | Any antibacterial  penicillin,  antibiotics, erythromycins, nitrofurantoins, sulphonamides, quinolones, cephalosporins, tetracyclines, other miscellaneousßlactams, aminoglycosides, antimycobacterial agents | Use, non-use | Computer assisted telephone interviews | One month before pregnancy to the end of the third month of pregnancy | Any Antibacterial  aOR 1.3 (1.0-1.6)  Penicillins  aOR 1.2 (0.8-1.8)  Erythromycins  aOR 0.9 (0.4-2.3)  Nitrofurantoins  aOR 1.6 (0.7-3.7)  Sulfonamides  aOR 0.8 (0.3-2.3)  Cephalosporins  aOR 0.8 (0.3-2.3) | Maternal age, BMI, race, education, smoking, alcohol, time from the estimated date of delivery to the interview, use of folic acid or multivitamins | 8 |
| David et al., 2014^[45]^  United Kingdom | Multicentre Case-control/  2006-2010/ultrasound scan/LB | 213 cases;  304 controls  Gastroschisis cases:59 | Folic acid | Use, non-use | Questionnaire | One month before through first month after conception,  first and second trimester | Folic acid  cOR 0.33 (0.18-0.63), |  | 5 |
| Feldkamp et al., 2011^[47]^  United States | Multicentre Case-control/ 1997-2005/birth defects surveillance system,  birth certificates or birth hospitals  (NBDPS)/LB, SB, ET | 694 Gastroschisis cases;  6,157 controls | Folic acid | Folic acid, DFE µg  48-385.2 µg (ref.)  385.3-634.4 µg  634.5-7283 µg | A modified Willett 58-item food frequency  questionare | First trimester | Folic acid intake  634.5-7283 µg  aOR 0.79 (0.2-1.02) | Maternal age, BMI, race/ethnicity, alcohol, education, smoking, energy intake (Kcal), time to interview, study center | 8 |
| Fisher et al., 2018^[49]^  United States | Multicentre Case-control/ 1997-2011/birth defects surveillance systems,  birth certificates or hospitals birth logs  (NBDPS)/LB, SB, ET | 17,038 cases;  11,477 controls  Gastroschisis cases:1,330 | Centrally-acting antiadrenergic agents,  β-blockers,  Renin-angiotensin system blockers,  calcium blockers,  diuretics, direct vasodilators | Use, non-use | Computer assisted telephone interview | One month before to third months after conception | Beta-blockers  cOR 0.5 (0.1-1.5)  Renin-angiotensin blockers  cOR 1.3 (0.3-4.5) | Maternal age, BMI, race/ethnicity, parity, pregestational diabetes, study center. (at least 5 exposed cases) | 8 |

| **Study,**  **Year, Country** | **Study design/ time/data source/outcome ascertainment** | **Sample^a^**  **Size** | **Exposure** | **Exposure definition** | **Exposure**  **assessment** | **Window of Exposure** | **Measures of effect**  **(95% CI)** | **Adjusted**  **variables** | **NOS**  **score^b^** |
| --- | --- | --- | --- | --- | --- | --- | --- | --- | --- |
| Furu et al., 2015^[51]^  Europe | Multicentre Cohort study/ 1996-2010/Nordic Nationwide Health Registries/LB | 36,772 cases;  2,266,857 live births  Gastroschisis cases: 424 | Selective serotonin reuptake inhibitors (SSRIs) or venlafaxine | Use, non-use | Nordic prescription registries | One month before to third months after conception | Any SSRIs  aOR 1.65 (0.87-3.15) | Maternal age, year of birth, birth order, smoking, maternal diabetes, using of other prescribed drugs | 8 |
| Garne et al., 2015^[52]^  Europe | Multicentre Case-control/ 1995-2010/EUROCAT congenital anomaly registries/LB, SB, ET | 16,803 cases;  9,578 malformed  controls 1  (chromosomal)  43,824 malformed controls2  (non – chromosomal)  Gastroschisis cases:615 | Any asthma medications  Inhaled ß_2_-agonists  Inhaled corticosteroids | Use, non-use | Obstetric/midwife records, medical records, records practitioner, maternity passports, maternal interviews | First trimester | Any Asthma Medications  Non-chromosomal  aOR 1.61 (1.04-2.50)  Chromosomal  aOR 1.76 (1.03-2.98)  Inhaled ß_2_-agonists  Non-chromosomal  aOR 1.89 (1.12-3.20)  Chromosomal  aOR 3.04 (1.53 to 6.06)  Inhaled corticosteroids  Non-chromosomal  aOR 0.59 (0.22 -1.60)  Chromosomal  aOR 0.33 (0.10 -1.11) | Maternal age, registry | 6 |
| van Gelder et al., 2014^[53]^  United States, Canada | Multicentre Case-control/ 1998-2010/Slone Birth Defects study, birth defects registries, hospital records, birth records/LB, SB | 5,568 cases;  7,253 controls  Gastroschisis cases:253 | Any antiadrenergic agents, angiotensin converting enzyme inhibitor, angiotensin II receptor blockers, calcium channel blockers, diuretics or direct vasodilator  Folic acid | Use, non-use | Mother interview | One month before pregnancy to the end of pregnancy  One month before to third months after conception | No pharmacological treated hypertension  aOR 0.3 (0.1-0.6)  Pre-eclampsia  aOR 0.8 (0.3 -1.9)  Folic acid  cOR 0.8 (0.8 to 0.9) | Centre, race/ethnicity, parity | 8 |
| Gilboa et al.,2009 ^[54]^  United States | Multicentre Case-control/ 1997-2003/birth defects  surveillance system, birth certificates or hospitals birth logs (NBDPS)/  LB, SB, ET | 9,078 cases;  4,982 controls  Gastroschisis  cases:473 | Any use of 54 different antihistamine agents collapsed into 14 analytic groups | Use, non-use | Computer assisted telephone interview | One month before pregnancy through the end of the first trimester | Any antihistamine  Non-Bayesian  aOR 1.17 (0.82-1.68)  Bayesian  aOR 1.26 (0.87-.82) | Maternal age, race/ ethnicity, education, entry into prenatal care, parity, household income, study center, folic acid use, alcohol, smoking, nausea and/or vomiting, respiratory infection | 8 |

| **Study,**  **Year, Country** | **Study design/ time/data source/outcome ascertainment** | **Sample^a^**  **Size** | **Exposure** | **Exposure definition** | **Exposure**  **assessment** | **Window of Exposure** | **Measures of effect (95% CI)** | **Adjusted**  **variables** | **NOS**  **score^b^** |
| --- | --- | --- | --- | --- | --- | --- | --- | --- | --- |
| Interrante et al., 2017^[57]^  USA | Multicentre Case-control/ 1997-2011/ birth defects surveillance systems,  birth certificates or hospitals birth logs | 16 450 cases;  5944 controls  Gastroschisis  cases:756 | Analgesic use | NSAIDs without opioids;  Opioids without NSAIDs;  Both opioids and NSAIDs;  Acetaminophen (Ref.) | Computer assisted telephone interview | One month before conception through the third month of pregnancy | NSAIDs without opioids  Exp= 401  aOR 1.6 (1.3 to 1.9)  Opioids without NSAIDs Exp=16  aOR 1.4 (0.8 to 2.6)  Both opioids and NSAIDs Exp=20  aOR 2.3 (1.2 to 4.2) | Maternal age, race/ethnicity, parity, education, alcohol, smoking, antibiotic use, study location, BMI estimate date of delivery. | 8 |
| Jenkins et al., 2014^[58]^  United States | Multicentre Case-control/ 1997-2003/birth defects surveillance systems,  birth certificates or hospitals birth logs  (NBDPS)/LB, SB, ET | 170 Gastroschisis cases;  1,484 controls | Folic acid | Use, non-use | Computer assisted telephone interview | One month before to third months after conception | Folic acid  cOR 0.79 (0.52-1.23) | Maternal age | 7 |
| Lam et al., 1999^[59]^  United States | Case-control (matched by age)/1988-1990/ California birth defects monitoring program (CBDMP), birth registry of the California Department of Vital Statistics/LB | 104 Gastroschisis cases;  200 controls | Asprin or Ibuprofen | Use, non-use | Mother interview | First trimester | Aspirin or ibuprofen  aOR 4.55 (0.99-20.81) | Maternal age, BMI, menarche to 1^st^ pregnancy, previous pregnancy losses, low alpha carotene, low total glutathione, high nitrosamine | 8 |
| Li et al., 2013^[60]^  United States | Case-control (matched by calendar year and birth region)/1998-2010/ Slone Epidemiology Center Birth defects registries/LB | 13,213 cases;  6,982 controls  Gastroschisis  cases:235 | Loratadine,  diphenhydramine, chlorpheniramine, doxylamine | Use, non-use | Computerized mother interview | First trimester | Diphenhydramine  aOR 0.8 (0.3-2.2) | Maternal age, race / ethnicity, education, household annual income, alcohol consumption, allergy and asthma in pregnancy, nausea/ vomiting, sleeping problems, respiratory infection, aspirin, corticosteroids, decongestants, acetaminophennon-steroidal anti-inflammatory drugs use | 8 |

| **Study,**  **Year, Country** | **Study design/ time/data source/outcome ascertainment** | **Sample^a^**  **Size** | **Exposure** | **Exposure definition** | **Exposure**  **assessment** | **Window of Exposure** | **Measures of effect (95% CI)** | **Adjusted**  **variables** | **NOS**  **score^b^** |
| --- | --- | --- | --- | --- | --- | --- | --- | --- | --- |
| Li et al., 2013^[61]^  United States | Case-control (matched by calendar year and birth region)/1998-2010/ Slone Epidemiology Center Birth defects registries/LB | 13,213 cases;  6,982 controls  Gastroschisis  cases:235 | Loratadine,  diphenhydramine, chlorpheniramine, doxylamine | Use, non-use | Computerized mother interview | First trimester | Diphenhydramine  aOR 0.8 (0.3-2.2) | Maternal age, race / ethnicity, education, household annual income, alcohol consumption, allergy and asthma in pregnancy, nausea/ vomiting, sleeping problems, respiratory infection, aspirin, corticosteroids, decongestants, acetaminophennon-steroidal anti-inflammatory use | 8 |
| Lin et al., 2008^[62]^  United States | Multicentre Case-control/ 1997-2002/birth defects surveillance systems,  birth certificates or hospitals birth logs  (NBDPS)/LB, SB, ET | 381 Gastroschisis cases; 4,121controls | Bronchodilators  Anti-inflammatories  Bronchodilators and  Anti-inflammatories | Use, non-use | Computer assisted telephone  interview | One month prior to conception through the third month of pregnancy | Bronchodilators  aOR 2.06 (1.19-3.59);  Anti-inflammatories  aOR 2.00 (0.88-4.51);  Bronchodilators and  Anti-inflammatories  aOR 2.69 (0.87-8.28); | Maternal age, ethnicity, education, smoking, folic acid, aspirin, methylene, ibuprofen  dioxymethamphetamine, amoxicillin, acetaminophen,  pseudoephedrine, phenylpropanolamine | 8 |
| Louik et al., 2013^[63]^  United States | Multicentre Case-control/ 2009-2011/Slone Epidemiology Center at Boston University birth hospital, defect registries and Vital statistic records/LB | 3,539 cases;  1,242 controls  Gastroschisis  cases:27 | H1N1 influenza vaccine in pregnancy | Exposed: received either the monovalent pH1N1 or the trivalent pH1N1 | Computer assisted telephone interview | First, second, and third trimesters | Exposed  aOR 0.83 (0.17-4.09) | Propensity score | 7 |
| Paranjothy et al., 2012^[65]^  UK | Multicentre Case-control (matched by age)/2007-2010/Fetal anomaly scan (FAS)/LB | 91 Gastroschisis cases;  217 controls | Folic acid | Folic acid intake  < 6 out first 12 weeks (Ref.)  ≥ 6 out first 12 weeks | Maternal interview | First trimester | Folic acid intake **≥**6 weeks  aOR 0.3 (0.1-0.7) | Maternal age, number of portions of fruit or vegetables eaten per week, BMI, NS-SEC classification, alcohol smoking, caffeine, folic acid, nausea or vomiting, changed partner. | 6 |
| Polen et al.,  2013^[66]^  United States | Multicentre Case-control/ 1997-2007/birth defects surveillance systems,  birth certificates or hospitals birth logs  (NBDPS)/LB, SB, ET | 19,043 cases; 8,002 controls  Gastroschisis cases:911 | Venlafaxine  Serotonin - norepinephrine reuptake inhibitors (SNRI) | Use, non-use | Computer assisted telephone interview | One month before conception through the first trimester of pregnancy | Venlafaxine  aOR 5.7 (1.8-15.9) | Maternal age, race/ethnicity | 8 |

| **Study,**  **Year, Country** | **Study design/ time/data source/outcome ascertainment** | **Sample^a^**  **Size** | **Exposure** | **Exposure definition** | **Exposure**  **assessment** | **Window of Exposure** | **Measures of effect (95% CI)** | **Adjusted**  **variables** | **NOS**  **score^b^** |
| --- | --- | --- | --- | --- | --- | --- | --- | --- | --- |
| Reefhuis et al., 2015^[69]^  United States | Multicentre Case-control /1997-2009/ birth defects surveillance systems, birth certificates or hospitals birth logs (NBDPS)/LB, SB, ET | 17,552 cases; 9,857 controls  Gastroschisis cases:18 | Selective Serotonin-Reuptake Inhibitors (SSRIs): citalopram, paroxetine, escitalopram,  sertraline, fluoxetine. | Use, non-use  Women who reported taking more than one type of SSRI  were included in the multiple SSRI category | Computer assisted telephone interviews | One month before pregnancy to the end of the third month of pregnancy | Paroxetine:  Posterior 1997-2009  aOR 2.5 (1.2 -4.8) | Maternal race or ethnicity, education, cigarette smoking, obesity. | 7 |
| Rittler et al.,  2015^[11]^  South America | Multicentre Case-control /1995-2010/Estudio Colaborativo Latino Americano de Malformaciones Congenitas (ECLAMC) maternity hospital network registry/LB, SB | 2,323 cases;  24,992 controls  Gastroschisis cases:472 | Medication  Sex hormones  Antacid | Use, non-use | Mother Interview | Any time during pregnancy | Medication  cOR 1.73 (1.14-2.63)  Sex hormones  cOR 1.94 (1.14-3.24)  Antacids  cOR 2.55 (1.17-5.54) |  | 5 |
| Siega-Riz et al., 2008^[71]^  United States | Multicentre Case-control /1997-2003/birth defects surveillance system,  birth certificates or birth hospitals (NBDPS)/LB, SB, ET | 464 Gastroschisis cases;  4,842 controls | Vasoconstrictor medication | Use, non-use | Computer assisted telephone interview | First trimester | Vasoconstrictor medication  cOR 1.4 (1.1-1.7) |  | 7 |
| Skarsgard et al., 2015^[12]^  Canada | Case-control/2006-2012/Canadian Pediatric Surgery Network database (CAPSNet) and Canadian Community Health Survey/LB, SB, ET | 692 Gastroschisis cases;  4,708 controls | Antidepressants | Use, non-use | Self-reported | During pregnancy | Antidepressants  aOR 4.04 (1.38 to 11.08) | Maternal age, smoking, alcohol, drug use, folic acid use, diabetes,  depressant use | 8 |
| Tinker et al., 2019^[72]^  United States | Multicentre Case-control/ 1997-2011/birth defects surveillance systems,  birth certificates or hospitals birth logs  (NBDPS)/LB | 18,136 cases;  11,614 controls  Gastroschisis cases:1,500 | Any benzodiazepine,  alprazolam,  clonazepam,  diazepam, lorazepam | Use, non-use | Computer assisted telephone interview | One month before conception through the third month of pregnancy | Any benzodiazepine  aOR 1.3 (0.7-1.7)  Alprazolam  cOR 09 (0.3-13.7) | Maternal age, race/ethnicity, antidepressant medication use | 7 |

| **Study,**  **Year, Country** | **Study design/ time/data source/outcome ascertainment** | **Sample^a^**  **Size** | **Exposure** | **Exposure definition** | **Exposure**  **assessment** | **Window of Exposure** | **Measures of effect (95% CI)** | **Adjusted**  **variables** | **NOS**  **score^b^** |
| --- | --- | --- | --- | --- | --- | --- | --- | --- | --- |
| Torfs et al., 1998^[73]^  United States | Multicentre Case-control (matched by age)/1988-1990/California Birth Defects Monitoring Program (CBDMP) registry, birth records of the California Department of Vital Statistics/LB | 55 Gastroschisis cases;  182 controls | Aspirin or Ibuprofen | Use, non-use | Maternal interview | 3 months before conception | Aspirin or Ibuprofen  aOR 4.1 (0.9-18.7) | Maternal age, low alpha carotene, low total glutathione, high nitrosamine, recreational drug, aspirin or ibuprofen | 7 |
| Wemakor et al., 2015^[76]^  Europe | Multicentre Case-control/ 1995-2009/EUROCAT congenital anomaly registries, hospital records/LB, SB, ET | 42,983 cases;  17,083 controls  Gastroschisis cases: 413 | Selective Serotonin-Reuptake Inhibitors  (SSRIs) combined and six SSRI components:  fluoxetine, citalopram,  paroxetine, sertraline,  fluvoxamine, escitalopram | Use, non-use | Obstetric/  midwife records prospectively collected or from or confirmed through maternal interviews after birth. | First trimester of pregnancy | Combined SSRIs  aOR 2.42 (1.10-5.29) | Registry | 5 |

Abbreviations: *CI*, confidence interval; *cOR*, crude odds ratio, *aOR*, adjusted odds ratio; *BMI*, body mass index; *NOS*, Newcastle-Ottawa Scale; *NBDPS*, National Birth Defects Prevention Study; *LB,* live births*; SB,* still births*; ET,* elective termination of pregnancy.

^a^Sample size represents the number of pregnancy episodes.

^b^The quality assessment of observational studies was based on the NOS score (range 0-9 stars) obtained from three criteria: selection (range 0-4 points); comparability (range 0-2 stars) and exposure (range 0-3 stars).

**Table S4. Newcastle-Ottawa Scale quality assessment of the studies included in meta-analysis**

| Author, year | Selection  (max = 4) | Comparability  (max = 2) | Exposure  (max = 3) | Overall quality score (max = 9) |
| --- | --- | --- | --- | --- |
| Draper et al., 2007^[44]^ | 4 | 2 | 3 | 9 |
| Feldkamp et al., 2010^[48]^ | 4 | 2 | 2 | 8 |
| Freitas et al., 2020^[50]^ | 4 | 1 | 1 | 6 |
| Given et al., 2017^[55]^ | 3 | 1 | 2 | 6 |
| Goodman al., 2019 ^[56]^ | 3 | 2 | 1 | 6 |
| Mac Bird et al., 2009^[15]^ | 4 | 2 | 2 | 8 |
| Martínez-Frías et al., 1997^[64]^ | 3 | 2 | 1 | 6 |
| Raitio et al., 2020^[67]^  Rebordosa et al., 2008^[68]^ | 3  4 | 1  2 | 2  3 | 6  9 |
| Robledo-Aceves at al., 2015^[70]^ | 3 | 2 | 1 | 6 |
| Torfs et al., 1996^[74]^ | 4 | 2 | 1 | 7 |
| Waller et al., 2010^[75]^ | 4 | 1 | 2 | 7 |
| Werler et al., 2018^[77]^ | 4 | 2 | 1 | 7 |
| Werler et al., 2009^[14]^ | 4 | 2 | 1 | 7 |
| Werler et al., 2003^[78]^ | 2 | 2 | 1 | 5 |
| Werler et al., 2002^[79]^ | 2 | 2 | 2 | 6 |
| Werler et al., 1992^[80]^ | 2 | 2 | 2 | 6 |
| Yau et al., 2013^[81]^ | 3 | 2 | 2 | 7 |


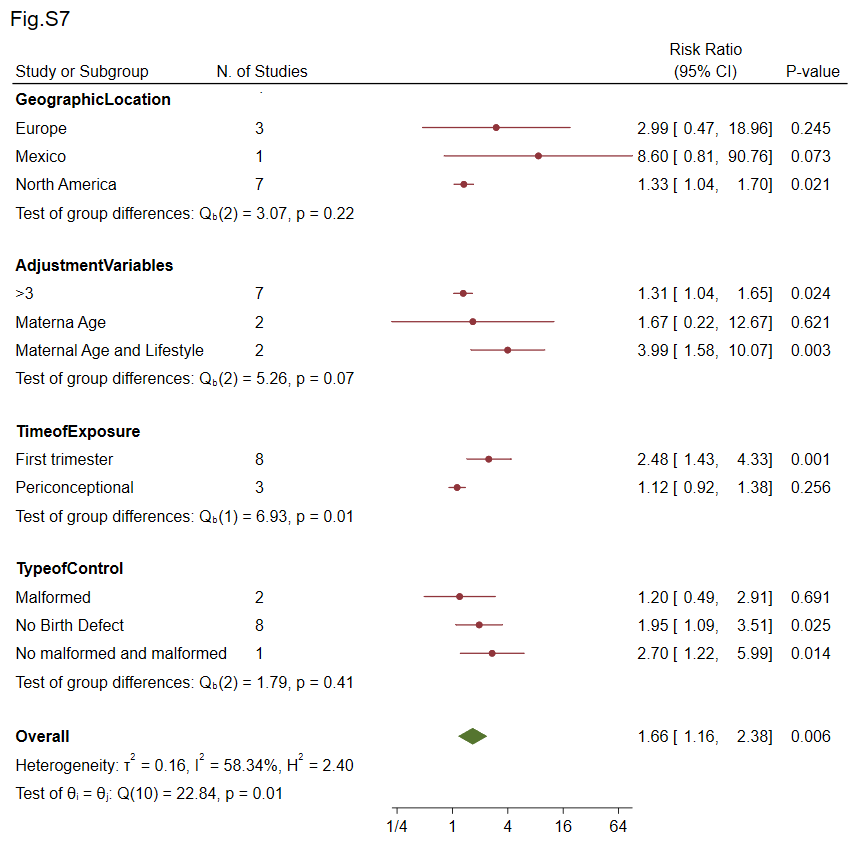


**Fig. S7** Random effect forest-plot of the association between aspirin use in pregnancy and gastroschisis, stratified by geographic location, adjustment variables, time of exposure and type of control


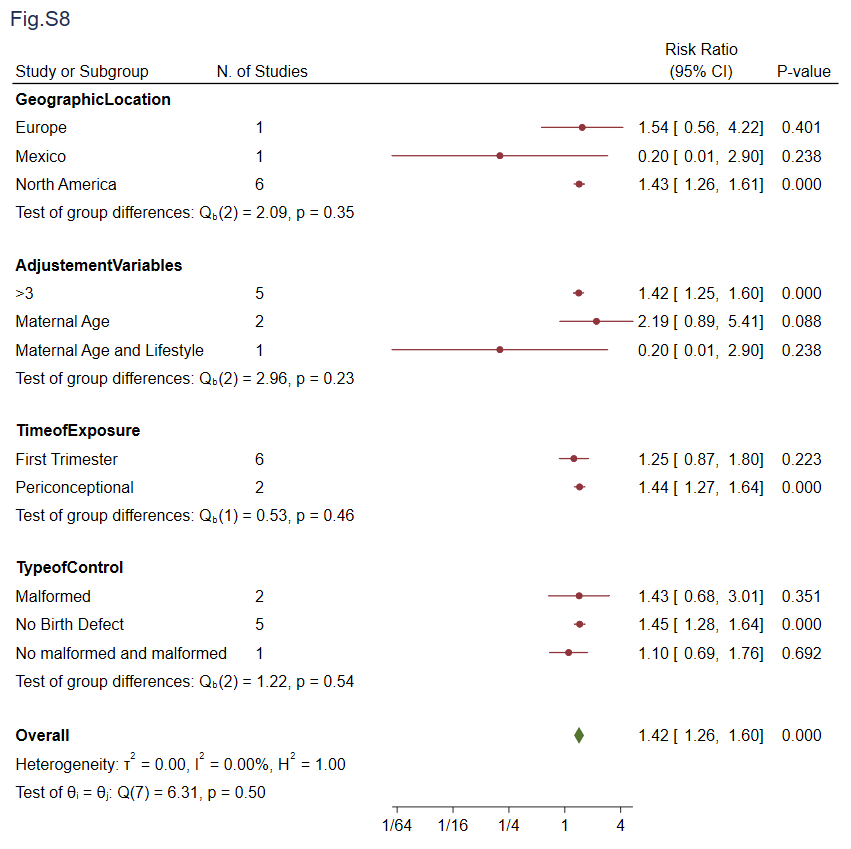


**Fig. S8** Random effect forest-plot of the association between ibuprofen use in pregnancy and gastroschisis, stratified by geographic location, adjustment variables, time of exposure and type of control


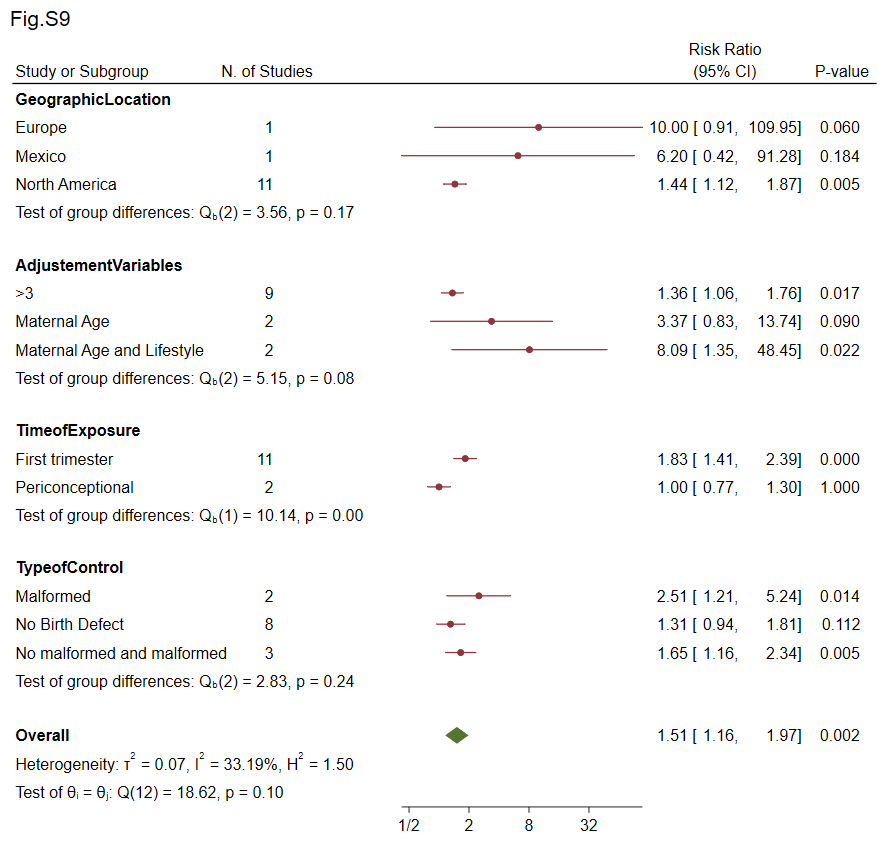


**Fig. S9** Random effect forest-plot of the association between decongestants use in pregnancy and gastroschisis, stratified by geographic location, adjustment variables, time of exposure and type of control


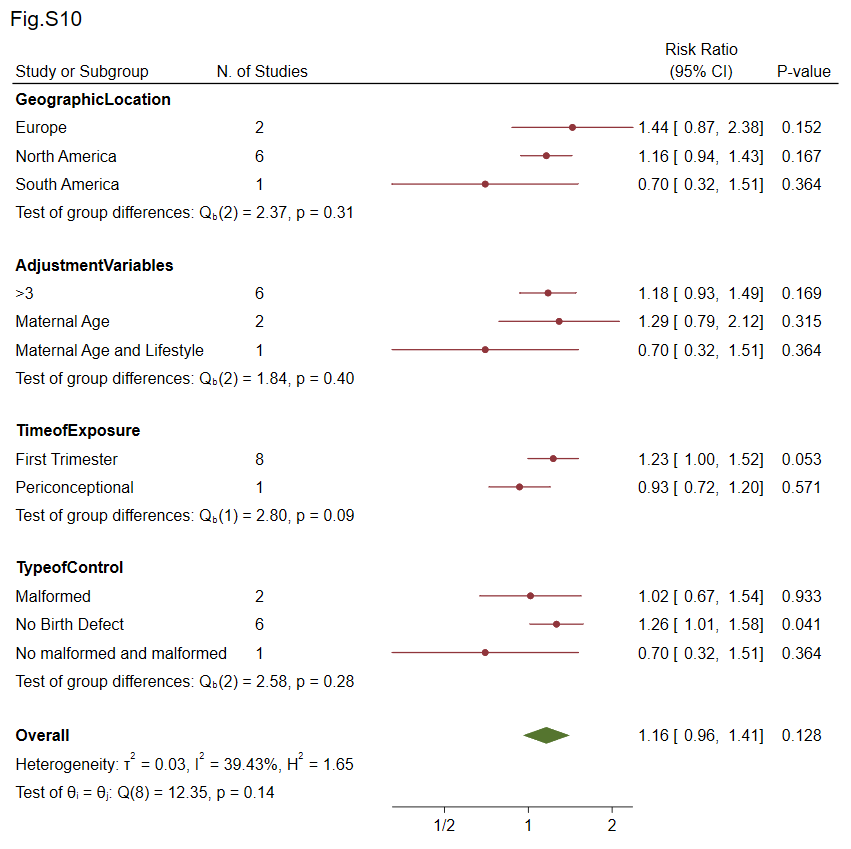


**Fig. S10** Random effect forest-plot of the association between paracetamol use in pregnancy and gastroschisis, stratified by geographic location, adjustment variables, time of exposure and type of control


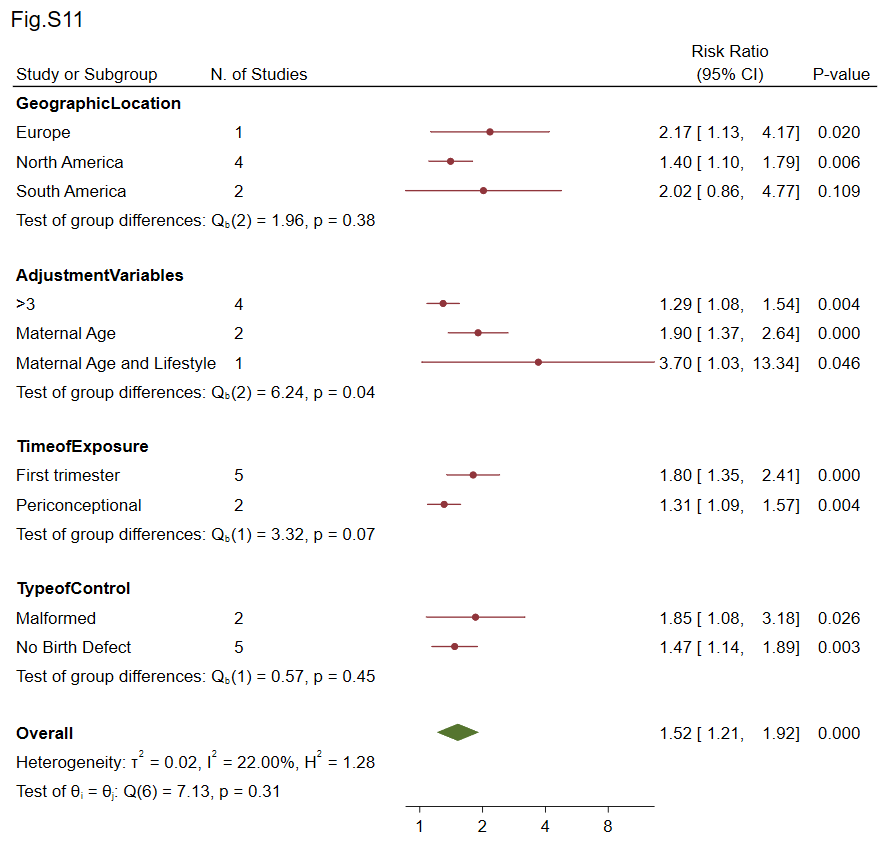


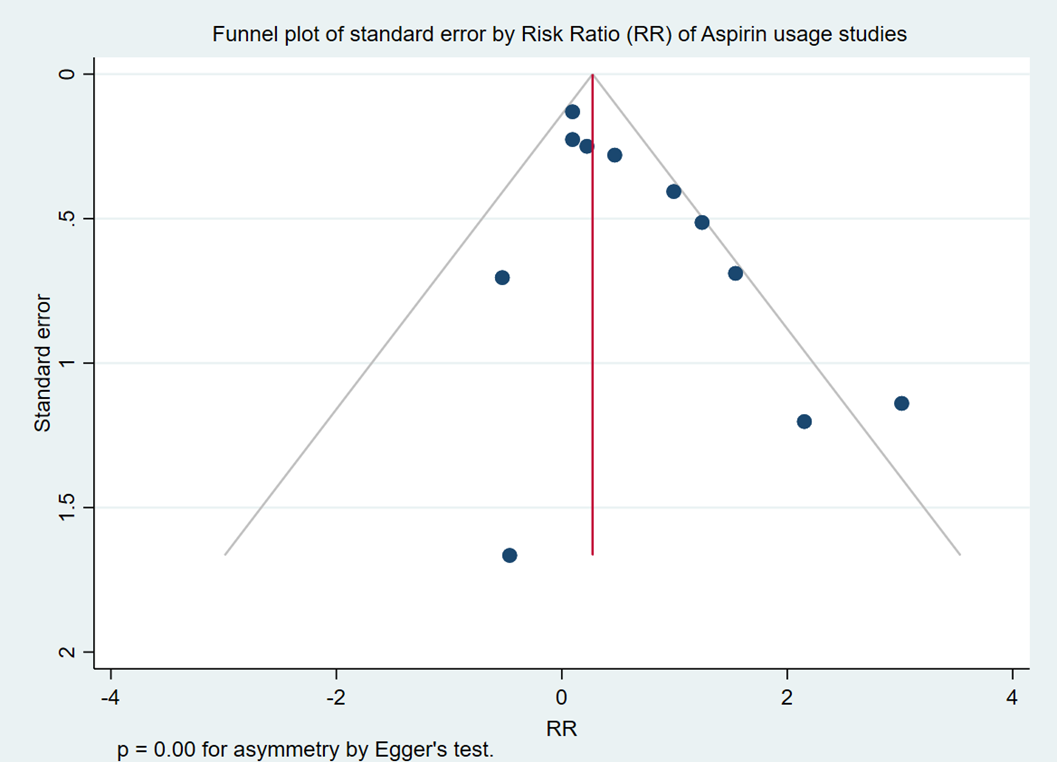


**Fig. S11** Random effect forest-plot of the association between oral contraceptive use in pregnancy and gastroschisis, stratified by geographic location, adjustment variables, time of exposure and type of control

Figure S7a

**Fig. S7a** Funnel plot for aspirin studies


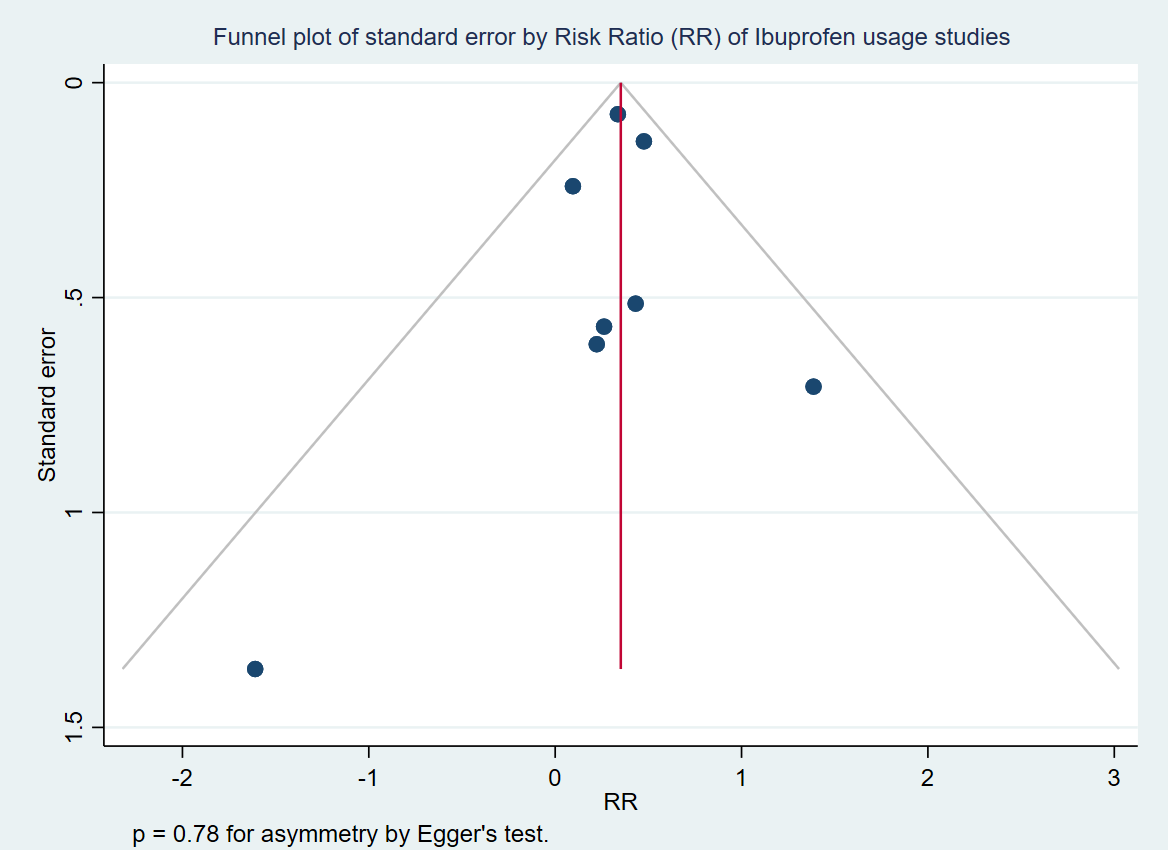


**Fig. S8a** Funnel plot for Ibuprofen studies


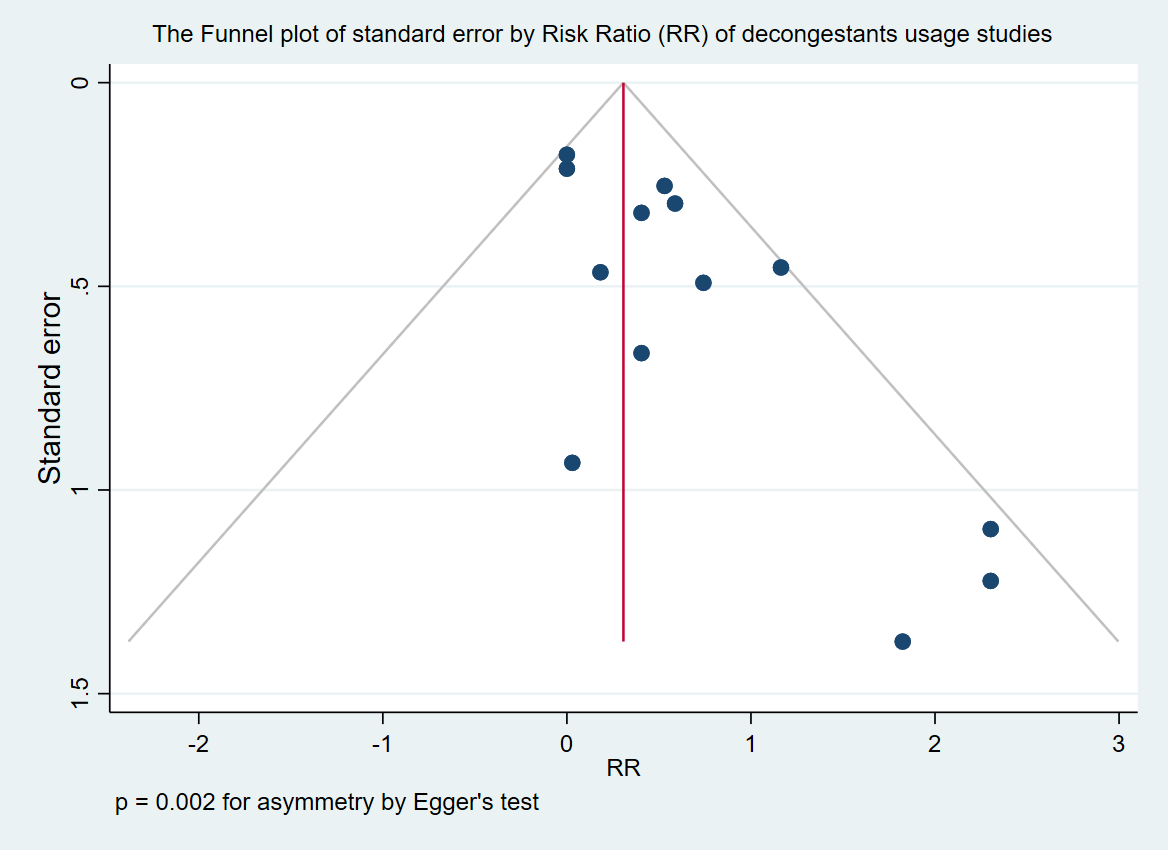


**Fig. S9a** Funnel plot for decongestant studies


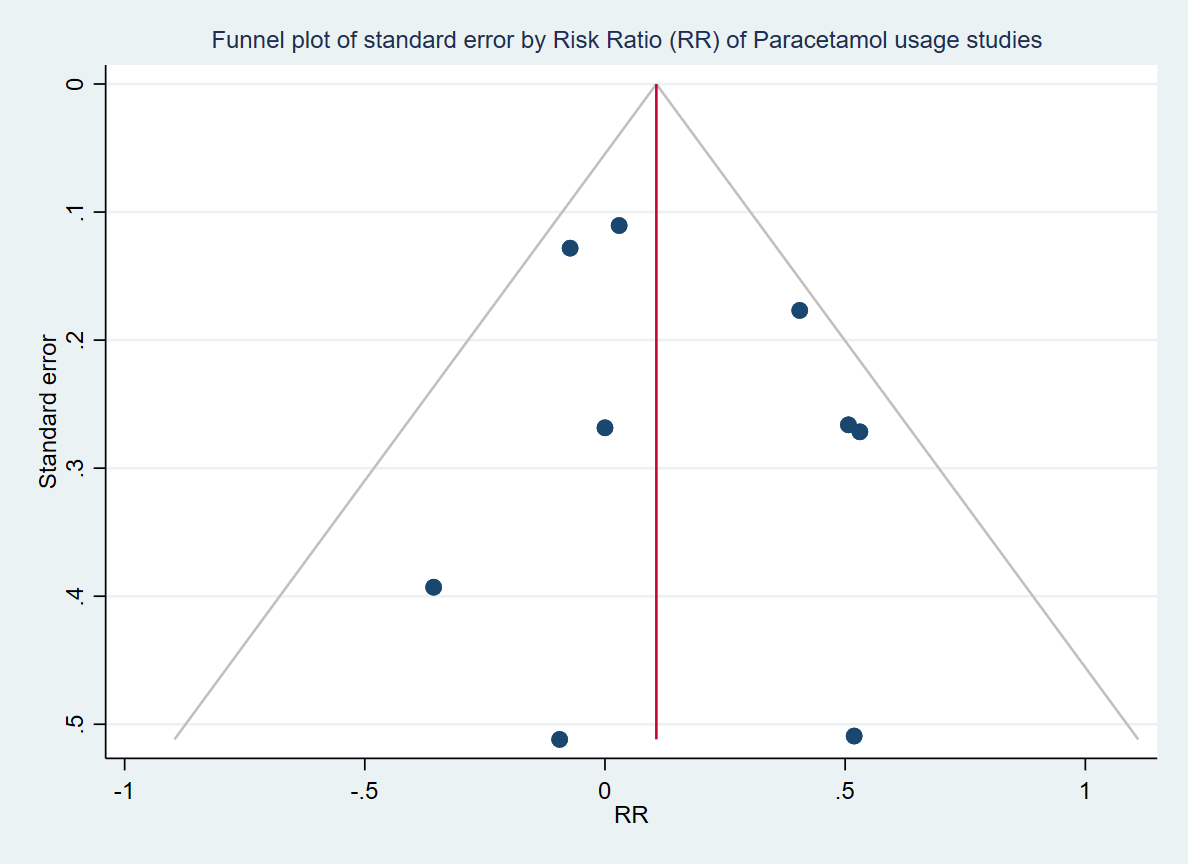


**Fig. S10a** Funnel plot for paracetamol studies


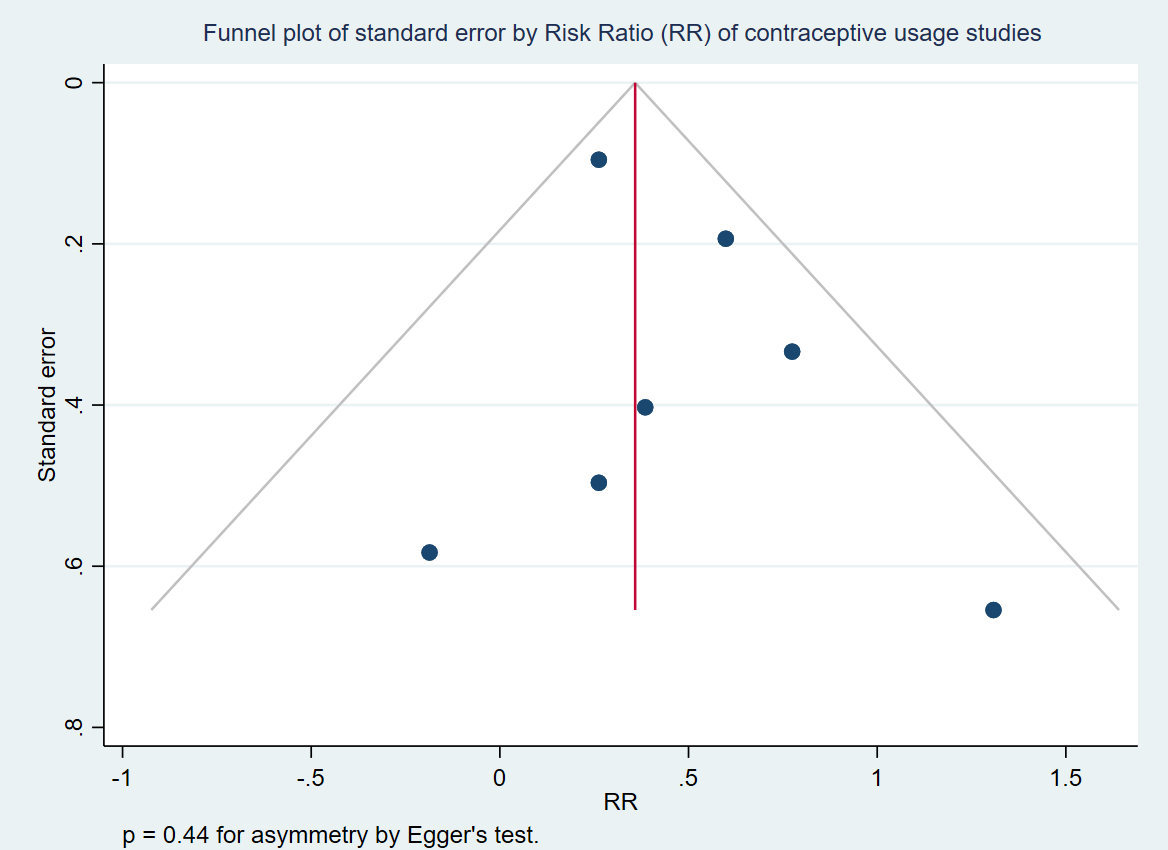


**Fig. S11a** Funnel plot for oral contraceptive studies

**References**

11. Rittler M, Campaña H, Ermini ML, Gili JA, Poletta FA, Pawluk MS, et al. Gastroschisis and young mothers: What makes them different from other mothers of the same age? Birth Defects Res A Clin Mol Teratol. 2015 Jun;103(6):536-43. doi: 10.1002/bdra.23374.

12. Skarsgard ED, Meaney C, Bassil K, Brindle M, Arbour L, Moineddin R; Canadian Pediatric Surgery Network (CAPSNet). Maternal risk factors for gastroschisis in Canada. Birth Defects Res A Clin Mol Teratol. 2015 Feb;103(2):111-8. doi: 10.1002/bdra.23349.

14. Werler MM, Mitchell AA, Moore CA, Honein MA; National Birth Defects Prevention Study. Is there epidemiologic evidence to support vascular disruption as a pathogenesis of gastroschisis? Am J Med Genet A. 2009 Jul;149A(7):1399-406. doi: 10.1002/ajmg.a.32897.

15. Mac Bird T, Robbins JM, Druschel C, Cleves MA, Yang S, Hobbs CA. Demographic and environmental risk factors for gastroschisis and omphalocele in the National Birth Defects Prevention Study. J Pediatr Surg. 2009;44:1546–51. doi: 10.1016/j.jpedsurg.2008.10.109..

34. Ahrens KA, Anderka MT, Feldkamp ML, Canfield MA, Mitchell AA, Werler MM. Antiherpetic medication use and the risk of gastroschisis: findings from the National Birth Defects Prevention Study, 1997-2007. Paediatr Perinat Epidemiol. 2013;27(4):340-5. doi: 10.1111/ppe.12064.

35. Ailes EC, Gilboa SM, Gill SK, Broussard CS, Crider KS, Berry RJ, et al. Association between antibiotic use among pregnant women with urinary tract infections in the first trimester and birth defects, National Birth Defects Prevention Study 1997 to 2011. Birth Defects Res A Clin Mol Teratol. 2016;106(11):940-949. doi: 10.1002/bdra.23570..

36. Alwan S, Reefhuis J, Rasmussen SA, Olney RS, Friedman JM. Use of selective serotonin-reuptake inhibitors in pregnancy and the risk of birth defects. N Engl J Med. 2007;356(26):2684-92. doi: 10.1056/NEJMoa066584.

37. Anderson KN, Dutton AC, Broussard CS, Farr SL, Lind JN, Visser SN, Ailes EC, Shapira SK, Reefhuis J, Tinker SC. ADHD Medication Use During Pregnancy and Risk for Selected Birth Defects: National Birth Defects Prevention Study, 1998-2011. J Atten Disord. 2020;24(3):479-489. doi: 10.1177/1087054718759753.

38. Anderson KN, Ailes EC, Lind JN, Broussard CS, Bitsko RH, Friedman JM, et al. Atypical antipsychotic use during pregnancy and birth defect risk: National Birth Defects Prevention Study, 1997-2011. Schizophr Res. 2020;215:81-88. doi: 10.1016/j.schres.2019.11.019.

39. Bitsko RH, Reefhuis J, Louik C, Werler M, Feldkamp ML, Waller DK, et al. Periconceptional use of weight loss products including ephedra and the association with birth defects. Birth Defects Res A Clin Mol Teratol. 2008;82(8):553-62. doi: 10.1002/bdra.20472.

40. Blotière PO, Raguideau F, Weill A, Elefant E, Perthus I, Goulet V, et al. Risks of 23 specific malformations associated with prenatal exposure to 10 antiepileptic drugs. Neurology. 2019;93(2):e167-e180. doi: 10.1212/WNL.0000000000007696.

41. Broussard CS, Rasmussen SA, Reefhuis J, Friedman JM, Jann MW, Riehle-Colarusso T, et al. Maternal treatment with opioid analgesics and risk for birth defects. Am J Obstet Gynecol. 2011;204(4):314.e1-11. doi: 10.1016/j.ajog.2010.12.039.

42. Carter TC, Druschel CM, Romitti PA, Bell EM, Werler MM, Mitchell AA. Antifungal drugs and the risk of selected birth defects. Am J Obstet Gynecol. 2008;198(2):191.e1-7. doi: 10.1016/j.ajog.2007.08.044.

43. Charlton BM, Mølgaard-Nielsen D, Svanström H, Wohlfahrt J, Pasternak B, Melbye M. Maternal use of oral contraceptives and risk of birth defects in Denmark: prospective, nationwide cohort study. BMJ. 2016;352:h6712. doi:10.1136/bmj.h6712

44. Crider KS, Cleves MA, Reefhuis J, Berry RJ, Hobbs CA, Hu DJ. Antibacterial medication use during pregnancy and risk of birth defects: National Birth Defects Prevention Study. Arch Pediatr Adolesc Med. 2009;163:978-85. doi:10.1001/archpediatrics.2009.188

45. David AL, Holloway A, Thomasson L, Syngelaki A, Nicolaides K, Patel RR, et al. A case-control study of maternal periconceptual and pregnancy recreational drug use and fetal malformation using hair analysis. PLoS One. 2014;9(10):e111038. doi: 10.1371/journal.pone.0111038.

46. Draper ES, Rankin J, Tonks AM, Abrams KR, Field DJ, Clarke M, Kurinczuk JJ. Recreational drug use: a major risk factor for gastroschisis? Am J Epidemiol. 2008;167(4):485-91. doi: 10.1093/aje/kwm335.

47. Feldkamp ML, Meyer RE, Krikov S, Botto LD. Acetaminophen use in pregnancy and risk of birth defects: findings from the National Birth Defects Prevention Study. Obstet Gynecol. 2010;115:109-115. doi:10.1097/AOG.0b013e3181c52616

48. Feldkamp ML, Carmichael SL, Shaw GM, Panichello JD, Moore CA, Botto LD. Maternal nutrition and gastroschisis: findings from the National Birth Defects Prevention Study. Am J Obstet Gynecol. 2011;204(5):404.e1-404.e10. doi: 10.1016/j.ajog.2010.12.053.

49. Fisher SC, Van Zutphen AR, Werler MM, Romitti PA, Cunniff C, Browne ML. Maternal antihypertensive medication use and selected birth defects in the National Birth Defects Prevention Study. Birth Defects Res. 2018;110(19):1433-1442. doi: 10.1002/bdr2.1372.

50. Freitas AB, Centofanti SF, Osmundo‐Junior GS, Rodrigues AS, Francisco RPV, Brizot ML. Risk factors for gastroschisis: A case–control study in a Brazilian population. Int J Gynecol Obstet. 2020;149: 347-353. https://doi.org/10.1002/ijgo.13135

51. Furu K, Kieler H, Haglund B, Engeland B, Selmer A, Stephansson R, et al. Selective serotonin reuptake inhibitors and venlafaxine in early pregnancy and risk of birth defects: population based cohort study and sibling design. BMJ. 2015;350:h2235. doi: 10.1136/bmj.h2235.

52. Garne E, Hansen AV, Morris J, Zaupper L, Addor MC, Barisic I, et al. Use of asthma medication during pregnancy and risk of specific congenital anomalies: A European case-malformed control study. J Allergy Clin Immunol. 2015;136(6):1496-1502.e7. doi: 10.1016/j.jaci.2015.05.043.

53. van Gelder MM, Van Bennekom CM, Louik C, Werler MM, Roeleveld N, Mitchell AA. Maternal hypertensive disorders, antihypertensive medication use, and the risk of birth defects: a case-control study. BJOG. 2015;122(7):1002-9. doi: 10.1111/1471-0528.13138.

54. Gilboa SM, Strickland MJ, Olshan AF, Werler MM, Correa A. Use of antihistamine medications during early pregnancy and isolated major malformations. Birth Defects Res A Clin Mol Teratol. 2009;85(2):137-50. doi: 10.1002/bdra.20513

55. Given JE, Loane M, Garne E, Nelen V, Barisic I, Randrianaivo H, et al. Gastroschisis in Europe - A Case-malformed-Control Study of Medication and Maternal Illness during Pregnancy as Risk Factors. Paediatr Perinat Epidemiol. 2017;31(6):549-559. doi: 10.1111/ppe.12401.

56. Goodman JR, Peck JD, Landmann A, Williams M, Elimian A. An evaluation of nutritional and vasoactive stimulants as risk factors for gastroschisis: a pilot study. J Matern Fetal Neonatal Med. 2019;32(14):2346-2353. doi: 10.1080/14767058.2018.1433657.

57. Howley MM, Papadopoulos EA, Van Bennekom CM, Van Zutphen AR, Carmichael SL, Munsie JW, et al. Asthma Medication Use and Risk of Birth Defects: National Birth Defects Prevention Study, 1997-2011. J Allergy Clin Immunol Pract. 2020;8(10):3490-3499.e9. doi: 10.1016/j.jaip.2020.07.033.

58. Interrante JD, Ailes EC, Lind JN, Anderka M, Feldkamp ML, Werler MM, et al. Risk comparison for prenatal use of analgesics and selected birth defects, National Birth Defects Prevention Study 1997-2011. Ann Epidemiol. 2017;27(10):645-653.e2. doi: 10.1016/j.annepidem.2017.09.003..

59. Jenkins MM, Reefhuis J, Gallagher ML, Mulle JG, Hoffmann TJ, Koontz DA, et al. Maternal smoking, xenobiotic metabolizing enzyme gene variants, and gastroschisis risk. Am J Med Genet A. 2014;164A(6):1454-63. doi: 10.1002/ajmg.a.36478.

60. Lam PK, Torfs CP, Brand RJ. A low pregnancy body mass index is a risk factor for an offspring with gastroschisis. Epidemiology. 1999;10(6):717-21. PMID: 10535786.

61. Li Q, Mitchell AA, Werler MM, Yau WP, Hernández-Díaz S. Assessment of antihistamine use in early pregnancy and birth defects. J Allergy Clin Immunol Pract. 2013;1(6):666-74.e1. doi: 10.1016/j.jaip.2013.07.008. Epub 2013 Sep 12. PMID: 24565715; PMCID: PMC4140658.

62. Lin S, Munsie JP, Herdt-Losavio ML, Bell E, Druschel C, Romitti PA, et al. Maternal asthma medication use and the risk of gastroschisis. Am J Epidemiol. 2008;168(1):73-9. doi: 10.1093/aje/kwn098..

63. Louik C, Ahrens K, Kerr S, Pyo J, Chambers C, Jones KL, et al. Risks and safety of pandemic H1N1 influenza vaccine in pregnancy: exposure prevalence, preterm delivery, and specific birth defects. Vaccine. 2013;31(44):5033-40. doi: 10.1016/j.vaccine.2013.08.096.

64. Martínez-Frías ML, Rodríguez-Pinilla E, Prieto L. Prenatal exposure to salicylates and gastroschisis: a case-control study. Teratology. 1997;56:241-3. doi:10.1002

65. Paranjothy S, Broughton H, Evans A, Huddart S, Drayton M, Jefferson R, et al. The role of maternal nutrition in the aetiology of gastroschisis: an incident case-control study. Int J Epidemiol. 2012;41(4):1141-52. doi: 10.1093/ije/dys092. Epub 2012 Jul 13. PMID: 22798661.

66. Polen KN, Rasmussen SA, Riehle-Colarusso T, Reefhuis J. Association between reported venlafaxine use in early pregnancy and birth defects, national birth defects prevention study, 1997-2007. Birth Defects Res A Clin Mol Teratol. 2013;97(1):28-35. doi: 10.1002/bdra.23096.

67. Raitio A, Tauriainen A, Leinonen MK, Syvänen J, Kemppainen T, Löyttyniemi E, et al. Maternal risk factors for gastroschisis: A population-based case-control study. Birth Defects Res. 2020;112(13):989-995. doi: 10.1002/bdr2.1703.

68. Rebordosa C, Kogevinas M, Horváth-Puhó E, Nørgård B, Morales M, Czeizel AE, et al. Acetaminophen use during pregnancy: effects on risk for congenital abnormalities. Am J Obstet Gynecol. 2008;198:178.e1-178.e1787. doi:10.1016/j.ajog.2007.08.040

69. Reefhuis J, Devine O, Friedman JM, Louik C, Honein MA. Specific SSRIs and birth defects: Bayesian analysis to interpret new data in the context of previous reports. BMJ. 2015;351:h3190. doi:10.1136/bmj.h3190

70. Robledo-Aceves M, Bobadilla-Morales L, Mellín-Sánchez EL, Corona-Rivera A, Pérez-Molina JJ, Cárdenas-Ruiz Velasco JJ, et al. Prevalence and risk factors for gastroschisis in a public hospital from west México. Congenit Anom (Kyoto). 2015;55(2):73-80. doi: 10.1111/cga.12087.

71. Siega-Riz AM, Herring AH, Olshan AF, Smith J, Moore C. The joint effects of maternal prepregnancy body mass index and age on the risk of gastroschisis. Paediatr Perinat Epidemiol. 2008;23:51-7. doi: 10.1111/j.1365-3016.2008.00990

72. Tinker SC, Reefhuis J, Bitsko RH, Gilboa SM, Mitchell AA, Tran EL, et al. Use of benzodiazepine medications during pregnancy and potential risk for birth defects, National Birth Defects Prevention Study, 1997-2011. Birth Defects Res. 2019;111(10):613-620. doi: 10.1002/bdr2.1497.

73. Torfs CP, Lam PK, Schaffer DM, Brand RJ. Association between mothers' nutrient intake and their offspring's risk of gastroschisis. Teratology. 1998;58:241-50. doi:10.1002

74. Torfs CP, Katz EA, Bateson TF, Lam PK, Curry CJ. Maternal medications and environmental exposures as risk factors for gastroschisis. Teratology. 1996;54:84-92. doi:10.1002

75. Waller DK, Gallaway MS, Taylor LG, Ramadhani TA, Canfield MA, Scheuerle A, et al. Use of oral contraceptives in pregnancy and major structural birth defects in offspring. Epidemiology. 2010;21(2):232-9. doi: 10.1097/EDE.0b013e3181c9fbb3.

76. Wemakor A, Casson K, Garne E, Bakker M, Addor MC, Arriola L, et al. Selective serotonin reuptake inhibitor antidepressant use in first trimester pregnancy and risk of specific congenital anomalies: a European register-based study. Eur J Epidemiol. 2015;30(11):1187-98. doi: 10.1007/s10654-015-0065-y.

77. Werler MM, Guéry E, Waller DK, Parker SE. Gastroschisis and Cumulative Stressor Exposures. Epidemiology. 2018;29(5):721-728. doi: 10.1097/EDE.0000000000000860.

78. Werler MM, Sheehan JE, Mitchell AA. Association of vasoconstrictive exposures with risks of gastroschisis and small intestinal atresia. Epidemiology. 2003;14:349-54.

79. Werler MM, Sheehan JE, Mitchell AA. Maternal medication use and risks of gastroschisis and small intestinal atresia. Am J Epidemiol. 2002;155(1):26-31. doi:10.1093/aje/155.1.26

80. Werler MM, Mitchell AA, Shapiro S. First trimester maternal medication use in relation to gastroschisis. Teratology. 1992;45:361-7. doi:10.1002/tera.1420450407

81. Yau WP, Mitchell AA, Lin KJ, Werler MM, Hernández-Díaz S. Use of decongestants during pregnancy and the risk of birth defects. Am J Epidemiol. 2013;178:198-208. doi:10.1093/aje/kws427
